# Nanodomain Control in Carbon Molecular Sieve Membranes via Nanomaterial Footprinting

**DOI:** 10.1002/smsc.202300162

**Published:** 2023-12-03

**Authors:** Rifan Hardian, Mahmoud. A. Abdulhamid, Gyorgy Szekely

**Affiliations:** ^1^ Advanced Membranes and Porous Materials Center Physical Science and Engineering Division (PSE) King Abdullah University of Science and Technology (KAUST) Thuwal 23955-6900 Saudi Arabia; ^2^ Chemical Engineering Program Physical Science and Engineering Division (PSE) King Abdullah University of Science and Technology (KAUST) Thuwal 23955-6900 Saudi Arabia

**Keywords:** carbon molecular sieves, composite membranes, fullerenols, nano-Fourier-transform infrared (nano-FTIRs), polymers of intrinsic microporosities

## Abstract

Carbon molecular sieve (CMS) membranes, fabricated via pyrolysis, are attracting attention owing to their stability under harsh environments, including high temperatures, organic media, and extreme pH. Herein, the fabrication of composite CMS (CCMS) membranes by incorporating sphere‐shaped C_60_(OH) and ellipsoid‐shaped C_70_(OH) fullerenol nanomaterials into intrinsically microporous 4,4′‐(hexafluoroisopropylidene) diphthalic anhydride 3,3′‐dimethyl‐naphthidine polyimide is reported. The encapsulation of the nanomaterials by the polymer matrix, their chemical footprint, and the variation in the local chemistry of the pyrolyzed membranes are successfully revealed via nanodomain analysis using nano‐Fourier‐transform infrared spectroscopy. The incorporation of fullerenol nanomaterials into CMS membranes can induce the formation of fractional free volume upon pyrolysis, which can translate into molecular sieving enhancement. The effects of the concentration and geometrical shape of the fullerenol nanomaterials are successfully correlated with the membrane separation performance. The CCMS membranes demonstrate excellent stability and pharmaceutical and dye separation performance in organic media. Herein, nanodomain control is pioneered in CCMS membranes via nanomaterial footprinting to induce porosity during pyrolysis and subsequent control molecular sieving performance.

## Graphical abstract

Synergetic effects of pyrolysis and fullerenol incorporation on the properties and performance of integrally skinned asymmetric composite carbon molecular sieve (ISA‐CCMS) membranes are reported. The design methodology builds on five essential pillars: 1) the geometrical aspects of nanomaterials, 2) nanomaterial dispersibility, 3) heat‐induced phase transformation of nanomaterials, 4) encapsulation of nanomaterials by polymer matrices, and 5) nanodomain footprinting and local chemical analysis of the ISA‐CCMS membrane.

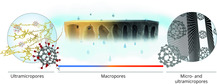

## 1 Introduction

The development of new materials is necessary to enable technological advancements. As there is almost unlimited potential for synthesizing new materials and combining existing materials for various applications, the rational design of material systems is essential to afford desired functionalities. Rational design concepts consider the properties of each material when different materials are combined to create a composite. This material design concept is essential for all fields, including energy‐efficient membrane separation. Membrane materials that exhibit robustness, resistance toward harsh environments, and long‐term stability are attractive, particularly for the oil and petrochemical industry, food and bioproducts industry, fine chemistry, and pharmaceutical industry.^[^ ^1^ ^]^ Therefore, various approaches have been explored to improve membrane stability, including crosslinking,^[^ ^2^, ^3^ ^]^ metal–polymer coordination,^[^ ^4^ ^]^ surface modification,^[^ ^5^ ^]^ and thermal annealing.^[^ ^6^ ^]^

An emerging strategy found to be effective in fabricating robust membranes is pyrolysis, which converts polymer membranes into carbon molecular sieve (CMS) membranes.^[^ ^7^ ^]^ In CMS membranes, hierarchical micropores (lower than 20 Å) are distributed bimodally.^[^ ^8^ ^]^ Ultramicropores (lower than 7 Å), which are formed by the slits between consecutive graphite‐like flakes or sheets, have the right sizes to separate small molecules, including ions, water, and even organic solvents. Meanwhile, micropores (7–20 Å), which exist as void spaces between stacked sheets, facilitate efficient pathways enabling molecular permeabilities.

Compared with pristine membranes, pyrolyzed membranes undergo structural rearrangement and pore shrinkage, which improves selectivity. Because of the remarkable selectivity of CMS membranes in the field of gas separation, their application has broadened to the field of liquid separation, including aqueous and organic solvent nanofiltration and forward and reverse osmosis.^[^ ^9^, ^10^, ^11^, ^12^, ^13^ ^]^ Although CMS membranes exhibit higher rejection profiles, they display lower liquid permeability than noncarbonized membranes because of the densification of polymer chain packing upon pyrolysis.^[^ ^7^ ^]^ Therefore, in this study, we propose a new rational design in preserving the spaces between the chain packing by incorporating nanomaterials into a polymer matrix for preparing composite membranes.

The advantages of composite membranes, particularly for liquid separation, have been reported in many studies.^[^ ^14^, ^15^, ^16^ ^]^ Particularly, composite CMS (CCMS) membranes refer to pyrolyzed composite membranes consisting of a polymer matrix and a nanomaterial filler. This type of membrane is also referred to as hybrid CMS or mixed‐matrix CMS.^[^ ^17^, ^18^ ^]^ However, the CMS field still lacks a fundamental understanding of the local chemical variability of composite membranes, nanomaterial encapsulation by the polymer matrix, phase transformation induced by pyrolysis, and the final chemical footprint of nanomaterials.

Although pyrolysis has been shown to modify the chemistry of materials,^[^ ^7^ ^]^ the chemical footprints of pyrolyzed materials, which are particularly important for nanocomposite materials, such as CCMS membranes, are yet to be understood. Moreover, in‐depth studies on the chemical inhomogeneity of membranes at the nanodomain level have never been reported. Further, standard attenuated‐total‐reflection Fourier‐transform infrared (ATR‐FTIR) spectroscopy, which has been widely used to reveal the chemical information of membranes at the macroscopic level, fails to access local information at the nanodomain level due to its limited spatial resolution.^[^ ^19^, ^20^, ^21^ ^]^

Thus, for the first time, we propose nano‐FTIR spectroscopy for chemical footprinting and chemical inhomogeneity analysis at the nanodomain of integrally skinned asymmetric CCMS (ISA‐CCMS) membranes. The nanoscale spatial resolution of nano‐FTIR enables probing local^[^ ^22^ ^]^ and subsurface^[^ ^23^, ^24^, ^25^, ^26^ ^]^ chemical information, which we exploit to study nanomaterial encapsulation by polymer matrices.

Herein, we present a new platform for designing CCMS membrane materials by considering five substantial aspects: 1) geometrical aspects of nanomaterials, 2) nanomaterial dispersibility, 3) heat‐induced phase transformation, 4) encapsulation of nanomaterials by polymer matrices, and 5) nanodomain footprinting and local chemical analysis of the CCMS membrane. Our rational design of the CCMS membrane fabrication provides a comprehensive understanding of the important aspects that contribute to the properties and performance of such membranes.

The 4,4′‐(hexafluoroisopropylidene) diphthalic anhydride 3,3′‐dimethyl‐naphthidine (6FDA‐DMN) was selected as the polymer matrix due to its kinked structure, which provides high fractional free volume (FFV) and surface area, excellent thermal stability, and high carbon content. Meanwhile, fullerenols were selected as fillers to preserve the membrane porosity owing to their high carbon content, well‐defined and variable molecular shape, and high dispersibility in organic solvents due to the abundance of hydroxyl groups.^[^ ^27^, ^28^ ^]^ Fullerenols can act as a polymer chain spacer and a cross‐linker to regulate the performance of membrane filtration.^[^ ^29^, ^30^, ^31^ ^]^ Two different types of fullerenols, i.e., C_60_(OH) and C_70_(OH), were used to study the effect of the molecular size and geometrical shape of the incorporated nanomaterials on the properties and molecular sieving performance of CCMS membranes. C_60_(OH) has the shape of a soccer ball, whereas C_70_(OH) resembles the shape of a rugby ball. Moreover, pyrolysis transformed C_60_(OH) into porous nanomaterials, whereas C_70_(OH) remained nonporous,^[^ ^32^ ^]^ enabling us to investigate the effects of nanomaterial porosity on CCMS properties. Furthermore, the effect of the fullerenol concentration on the nanofiltration performance of the CCMS membranes was also investigated.

**Figure 1:**
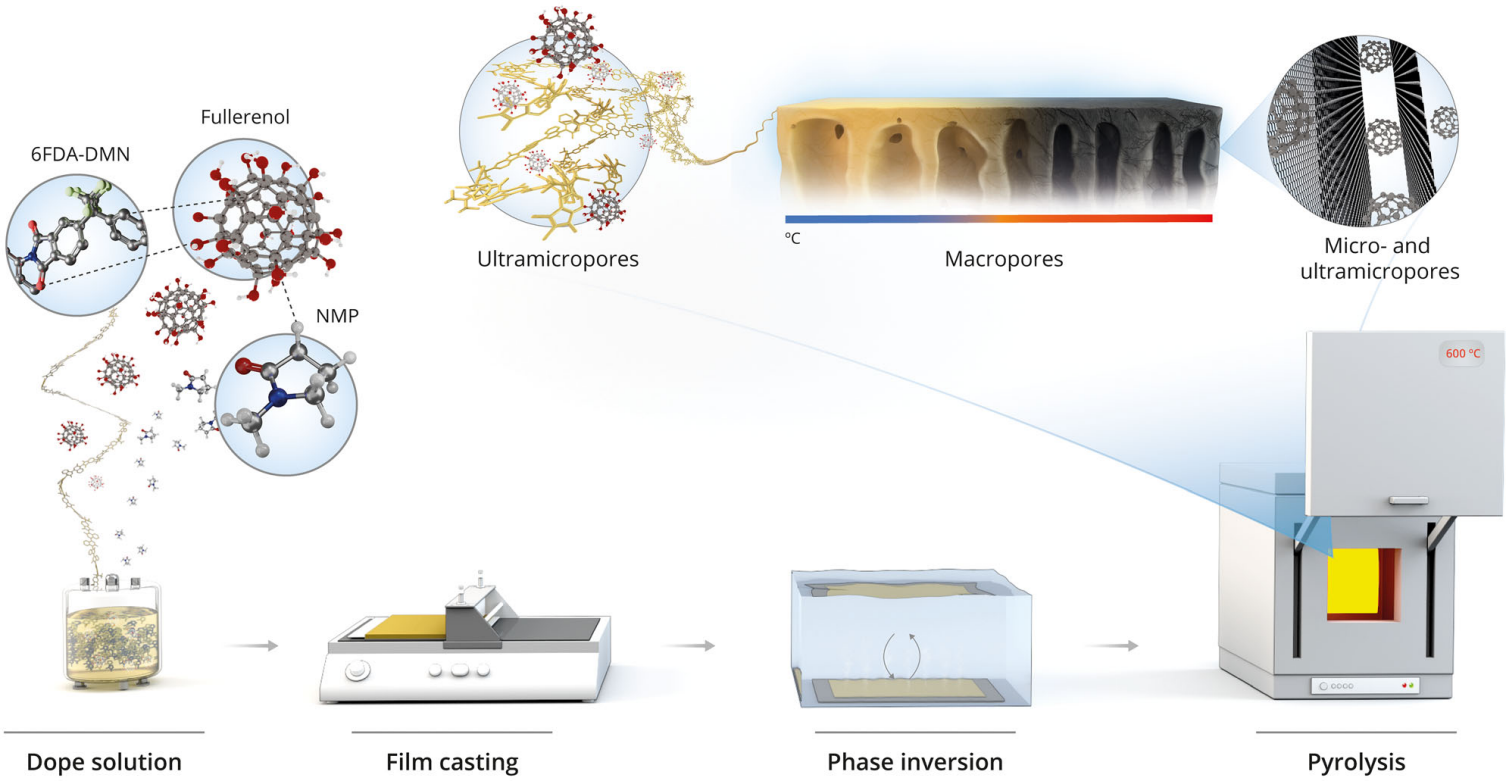
Schematic of the preparation of the integrally skinned asymmetric composite carbon molecular sieve membrane. Compared to the pristine composite membrane, a smaller macrovoid configuration is observed in the pyrolyzed membrane. Hydroxylated fullerene (fullerenols) transformed into nonhydroxylated nanomaterials upon pyrolysis and served as spacers in between carbonized polymeric chains.

The ISA‐CCMS configuration was chosen over the dense membrane configuration owing to its better suitability for liquid separations.^[^ ^7^ ^]^ Dense membranes typically have a lower porosity than other configurations, which is more suitable for gas separation. The ISA‐CCMS membranes were fabricated via phase inversion followed by pyrolysis (**Figure** **1**).

## 2 Results and Discussion

### 2.1 Polymer and Membrane Characterizations

The 6FDA‐DMN was prepared via a polycondensation reaction. The chemical structure of 6FDA‐DMN was confirmed via ^1^H nuclear magnetic resonance (NMR) (Figure S1, Supporting Information), ^19^F NMR (Figure S2, Supporting Information), and FTIR (Figure S3, Supporting Information). Increasing the polycondensation reaction temperature to 200 °C allowed the full imidization of the polyimide, as verified by the absence of carboxylic acid peaks in NMR after 10 ppm (Figure S1, Supporting Information) and through FTIR between 3000 and 3700 cm^−1^ (Figure S3, Supporting Information). The 6FDA‐DMN exhibited high thermal stability, with an onset decomposition temperature exceeding 450 °C. To afford solvent‐resistant membranes, we pyrolyzed ISA‐CCMS membranes at 600 °C.

**Figure 2:**
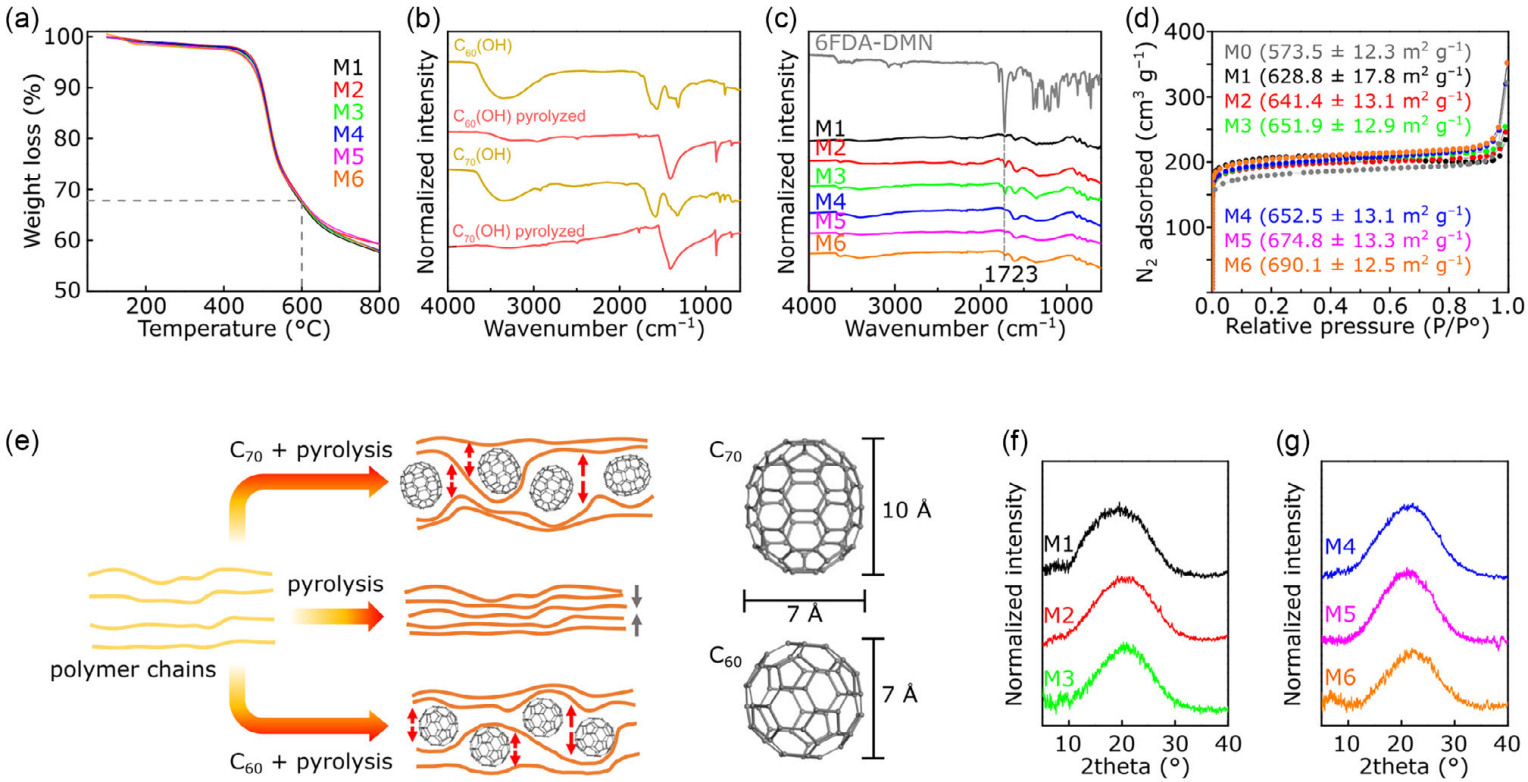
a) Thermogravimetric analysis curves of M1–M6 before pyrolysis. b) Fourier transform infrared (FTIR) spectra of fullerenol nanomaterials before and after pyrolysis. c) FTIR spectra of 6FDA‐DMN membranes before pyrolysis and M1–M6. d) Nitrogen adsorption isotherms and their Brunauer–Emmett–Teller values for M1–M6. e) Illustration of polymer chain packing upon pyrolysis. f,g) X‐ray diffraction patterns of M1–M3 (f) and M4–M6 (g).

The details of the membrane fabrication in this study are provided in Experimental Section. The ISA‐CCMS membranes derived from 6FDA‐DMN containing 1, 2, and 3 wt% C_60_(OH) nanomaterials in the dope solution were designated M1, M2, and M3, respectively. Similarly, the ISA‐CCMS membranes derived from 6FDA‐DMN containing 1, 2, and 3 wt% C_70_(OH) nanomaterials in the dope solution were designated M4, M5, and M6, respectively. The characteristics of the pristine membrane prior to pyrolysis have been reported in our previous publication.^[^ ^7^ ^]^ The incorporation of fullerenols into the 6FDA‐DMN matrices (i.e., M1–M6) did not influence the thermal behavior of the composite membranes relative to that of pristine 6FDA‐DMN (**Figure** **2**a). Meanwhile, FTIR analysis (Figure 2b) revealed structural changes upon pyrolyzing the C_60_(OH) and C_70_(OH) fullerenols. The disappearance of functional groups in pristine fullerenols (such as –OH, C=C, and C–OH at 775, 1600, and 1300 cm^−1^, respectively) was accompanied by the emergence of new functional groups corresponding to new bands at 877 and 1410 cm^−1^, which are typical for carbonate formation^[^ ^33^ ^]^ and the bending mode of covalent C–O bonds.^[^ ^34^ ^]^

**Figure 3:**
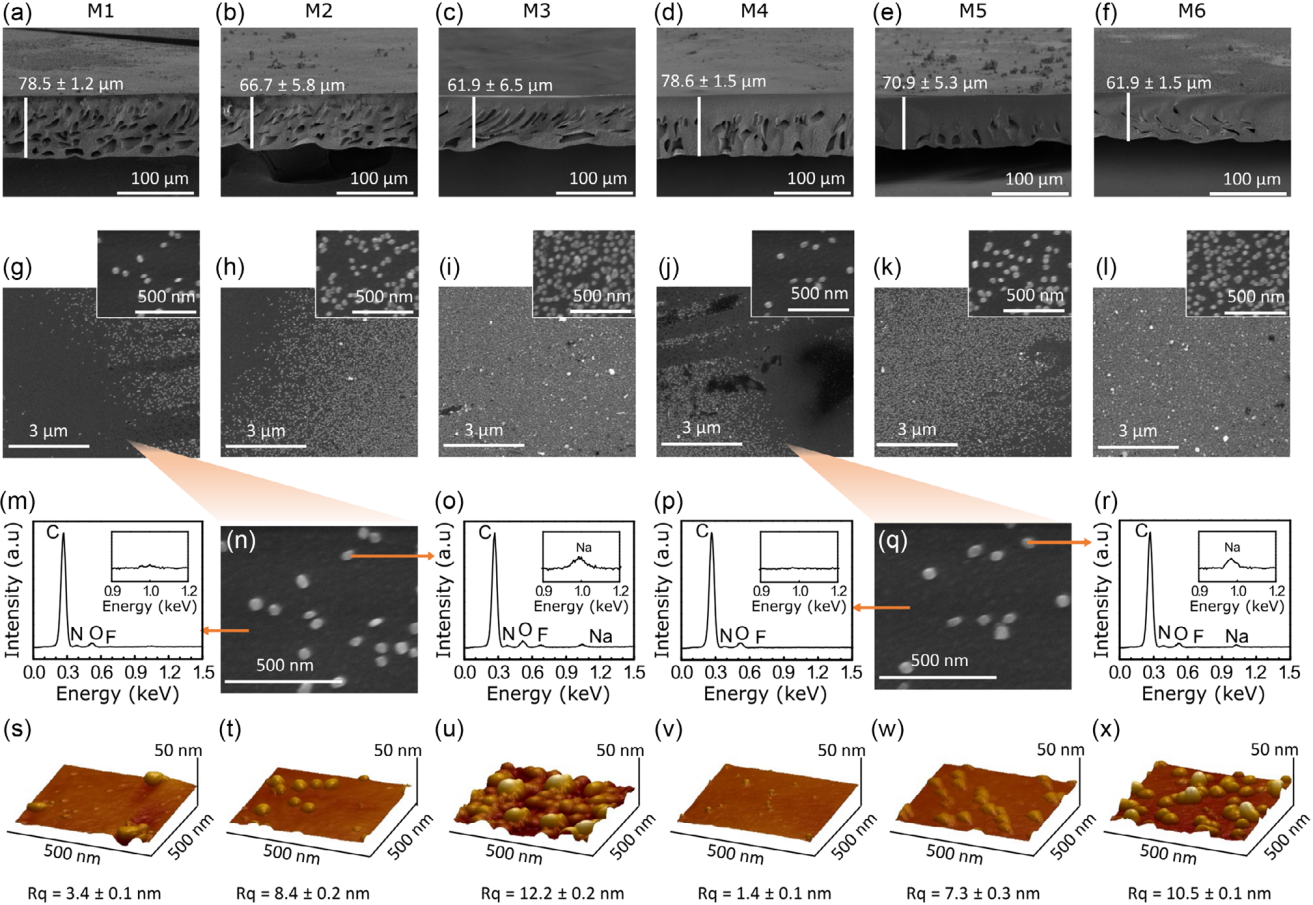
a–f) Scanning electron microscopy cross section and g–l) surface images of M1–M6, respectively. m–r) Energy‐dispersive X‐ray spectra of the polymer matrix and fullerenol on M1 (m–o) and M4 (p–r). s–x) Atomic force microscopy (AFM) images of M1–M6.

**Figure 4:**
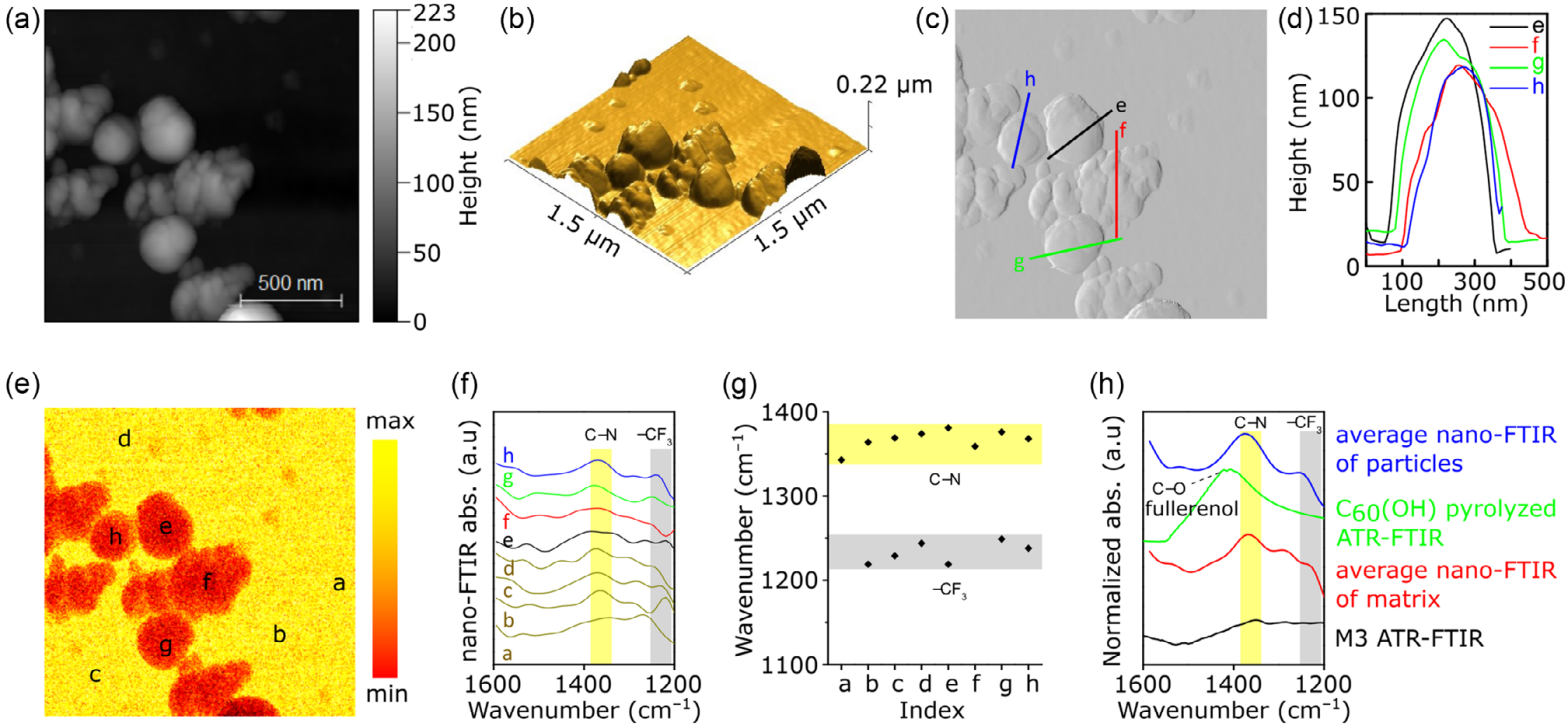
a) AFM height image and b) its 3D topography projection. c) Corresponding mechanical amplitude image and d) lateral and height dimensions of representative particles. e) Optical amplitude image with locations for nano‐FTIR measurement. f) Nano‐FTIR spectra of locations marked in the optical amplitude image. g) Variation of the spectra peak position corresponding to –CF_3_ and –CN functional groups. h) Comparison of the spectra collected via ATR‐FTIR and the average of nano‐FTIR.

The band corresponding to the polymer matrix (≈1723 cm^−1^ for C=O from 6FDA) was weakly observed in the FTIR spectra of the ISA‐CCMS membranes (Figure 2c). Meanwhile, characteristic bands for the pyrolyzed fullerenols (≈1410 cm^−1^) were not detected, although it was evidenced from the energy‐dispersive X‐ray (EDX) spectroscopy, which is further explained in **Figure** **3**. This observation can be attributed either to the low amount of fullerenol in the polymer matrix or to the encapsulation of fullerenol by the polymer matrix, which resulted in the vibration bands of 6FDA‐DMN dominating the FTIR spectra. Owing to the limitation of spatial resolution in standard ATR‐FTIR, it cannot reveal the chemical information of low nanomaterial concentration in the matrix, in particular when the nanomaterials are enveloped by the polymer matrix. Therefore, in the following section (**Figure** **4**), we provided nano‐FTIR analysis with nanoscale spatial resolution to reveal the nanodomain information. The functional groups of the 6FDA‐DMN polymer in the ISA‐CCMS membranes almost completely diminished upon pyrolysis, indicating substantial structural degradation in the polymer chains and the partial elimination of elements, such as oxygen, nitrogen, and fluorine.

Nitrogen adsorption isotherm data were collected to further investigate the effects of fullerenol intercalation on the porosity of ISA‐CCMS membranes (Figure 2d). Interestingly, the Brunauer–Emmett–Teller (BET) surface areas of all the studied ISA‐CCMS membranes exhibited values higher than 628.8 m^2^ g^−1^ (for M1), which were higher than those of the pristine ISA‐CMS membranes reported previously (575 m^2^ g^−1^).^[^ ^7^ ^]^ This indicated that the intercalation of fullerenol nanomaterials successfully increased the porosity of the ISA‐CCMS membranes by creating spaces between the polymer chains. A further increase in the C_60_ fullerenol concentration enhanced the BET surface areas even further (641.4 and 651.9 m^2^ g^−1^ for M2 and M3, respectively). The intercalation of C_70_ fullerenol (M4–M6) created higher BET surface areas (652.5–690.1 m^2^ g^−1^) than those for the intercalation of C_60_ fullerenol (M1–M3).

In addition to N_2_ sorption isotherm, we also performed CO_2_ sorption isotherm measurements (the isotherms and pore size distributions are presented in Figure S7, Supporting Information, and the data is tabulated in Table S1, Supporting Information). A CO_2_ molecule has a kinetic diameter of 3.3 Å, 10% smaller than the kinetic diameter of a N_2_ molecule (3.64 Å), thus enabling the penetration and diffusion of CO_2_ molecules through the ultramicropores. The CO_2_ sorption isotherm (Figure S7a, Supporting Information) showed that the uptake increased when the concentrations of the filler were increased. CCMS membrane containing C_70_ fullerenols as fillers (M4–M6) exhibited higher uptake than CCMS membrane containing C_60_ fullerenols (M1–M3). The pore size determined from CO_2_ sorption measurements showed both ultramicroporosity (centered around 6.0 Å) and microporosity (centered around 8.3 Å), which is characteristic of the CMS membranes.

The BET analyses confirmed two important findings. First, the incorporation of both the nanomaterials induced higher ISA‐CCMS porosity, indicating the enhancement of FFV regardless of the different pyrolysis‐induced transformations of C_60_(OH) (porous nanomaterial) and C_70_(OH) (nonporous nanomaterial). Second, the larger size of C_70_(OH) and its elongated‐shape‐induced higher ISA‐CCMS porosity than that of C_60_(OH). Hence, the molecular size and orientation of the intercalated nanomaterials influenced the arrangement of polymer packing.

The effects of pyrolysis and nanomaterial geometries on polymer chain packing are illustrated in Figure 2e. Upon pyrolysis, the shrinkage of the polymer shortened the distance (*d*‐spacing) between the polymer chains, indicating denser chain packing and enhanced tightness.^[^ ^7^ ^]^ In contrast, the intercalation of fullerenol nanomaterials affected the shrinkage of the polymer chains upon pyrolysis. Our results demonstrate that the porosity of the ISA‐CCMS membranes can be fine‐tuned by controlling the amount and type of nanomaterials.

Due to the amorphous nature of ISA‐CCMS, the change in the broad X‐ray diffraction (XRD) peaks representing the spacing alteration was not prominent (Figure 2f,g). Membranes M1–M6 exhibited amorphous characteristics with no peaks corresponding to the fullerenol nanomaterials, whose 2*θ* could be expected at ≈10° and 20°.^[^ ^32^ ^]^ The similarity of the XRD patterns in M1–M6 could be attributed to the addition of a low amount of fullerenols (1–3 wt%); hence, the XRD patterns were only dominated by the diffraction from the polymer matrix. Moreover, the small increase in the amount of fullerenol did not greatly affect the XRD patterns. Note that XRD analysis only reveals the general polymer arrangement. Therefore, local arrangements, such as the increased distance between polymer chains due to fullerenol intercalation, may not be precisely observed, especially when the XRD peak is dominated by a broad, amorphous peak.

Nanoindentation experiments showed that the CCMS membranes exhibited adequate mechanical properties, which did not seem to be influenced by the type and amount of the incorporated fullerenols (Table S1, Supporting Information). Although the obtained CCMS membranes were somewhat brittle as reported for other type of CMS membranes,^[^ ^7^, ^35^ ^]^ all the membranes showed sufficient mechanical stability to be used for organic solvent nanofiltration (OSN) at pressures as high as 10 bar.

The cross‐sectional morphology of M1–M6 displayed an ISA characteristic with a dense selective layer and macrovoid substructure (Figure 3a–f). The thickness of the membranes ranged from 62 to 78 μm. Additional cross‐sectional scanning electron microscopy (SEM) images with higher magnification to estimate the skin layer thickness of the pristine membrane and the CCMS membranes were provided in Figure S6, Supporting Information. The skin layer thickness of the pristine membrane prior to the conversion to CCMS membrane was estimated to be around 2.1 μm. After the conversion to CCMS, the skin layer thickness varied between 3 and 5 μm. The thicker skin layer is a result of the densification induced by the polymer rearrangement during pyrolysis at high temperatures. The surface morphology of the ISA‐CCMS membranes displayed the presence of nanomaterials on the membrane surface (Figure 3g–l). The spherical nanomaterials observed on the surfaces of the ISA‐CCMS membranes containing either C_60_(OH) or C_70_(OH) represented agglomerated, and not single‐molecule fullerenols. The size of a single‐molecule C_60_ fullerene was ≈7 Å with an internal diameter of 3.7 Å, whereas C_70_ fullerene had a dimension of 7 × 10 Å with an internal diameter of 4.6 × 3.7 Å.^[^ ^36^ ^]^ The sizes of single‐molecule C_60_ and C_70_ fullerene could not be observed via SEM. The presence of nanomaterials was more pronounced, and their distributions were more homogenous as the nanomaterials’ concentration increased (M1 < M2 < M3 and M4 < M5 < M6). Interestingly, we did not observe the fullerenol agglomerates on the cross section of the membranes (Figure S6, Supporting Information), which could indicate that the nanoparticles are dispersed in a smaller size. Further clarification of the presence of smaller size fullerenol nanoparticles are discussed in the next section on molecular sieving performance.

EDX spectroscopy was performed to elucidate the elemental analysis of the nanomaterials and polymer matrix (Figure 3m,o,p,r). Carbon (C), nitrogen (N), oxygen (O), and fluorine (F) were detected in the polymer matrix, whereas an additional peak of sodium (Na) was detected only in the nanomaterials. The detection of sodium in the nanomaterials ensured the identification of nanomaterials as fullerenols, which originated from the fullerenol synthesis procedure involving sodium hydroxide.^[^ ^37^ ^]^ Whereas the detection of fluorine in the nanomaterials indicated the coexistence of the polymer layer covering the nanomaterials surface.

Further, the existence of fullerenol nanomaterials on the membrane surfaces was characterized via atomic force microscopy (AFM) (Figure 3s–x). In line with the SEM analysis (Figure S5, Supporting Information), the size of the fullerenol nanomaterials determined by AFM was also ≈50 nm. The coverage of the membrane surface by fullerenol nanomaterials was more homogenous as the nanomaterials’ concentration increased. Accordingly, the roughness of the membranes also increased with increasing concentrations of nanomaterials. The roughness values of M1, M2, and M3 were 3.4, 8.4, and 12.2 nm, respectively. Meanwhile, the roughness values of M4, M5, and M6 were 1.4, 7.3, and 10.5 nm, respectively.

Although the characteristics of fullerenols were successfully identified via EDX analysis and AFM, it was not clear whether the fullerenol nanomaterials were fully enveloped by the polymer. During the EDX analysis, a high‐energy electron beam (20 keV) that could easily penetrate the thin layer of the polymer film and probe the nanomaterials beneath the polymer surface was applied. Meanwhile, extracting meaningful chemical information from the pyrolyzed membranes (ISA‐CCMS) was impossible using standard FTIR because of the low intensity of the spectra (Figure 2c). Moreover, the standard FTIR spectroscopy technique provides only average chemical information from a macroscopic level and fails to identify local information.

To overcome those challenges, a nano‐FTIR spectroscopy technique was applied to reveal the chemical information of the ISA‐CCMS membranes at the nanodomain level. Nano‐FTIR is a combined AFM and FTIR technique that allows the identification of the local chemistry of materials. Unlike EDX spectroscopy, low‐energy infrared in nano‐FTIR enables to probe the chemical information on the membrane surface.

In this study, M3 was selected as the representative material for in‐depth analysis via nano‐FTIR spectroscopy. The AFM height image and its corresponding 3D topography projection are shown in Figure 4a,b, respectively. For the nano‐FTIR study, we intentionally selected an area where larger agglomerated particles were observed to investigate whether the polymer matrix would still fully envelop the nanomaterials even though their size was large. The corresponding mechanical amplitude image and the nanomaterial dimensions are displayed in Figure 4c,d, respectively. In the selected regions, the agglomerated nanomaterials showed a lateral dimension of 200–300 nm, with part of the agglomerate (≈100–150 nm height) bulging out of the membrane.

In the optical image (Figure 4e), a strong material contrast between the pyrolyzed polymer matrix (yellow) and the fullerenol nanomaterials (red) could be observed. This contrast was related to the variation in the refractive index between the two components.^[^ ^22^ ^]^ The nano‐FTIR spectra (Figure 4f) were collected at selected locations, as marked in Figure 4e. Interestingly, the nano‐FTIR spectra in locations a–d appeared to be different, although these points were located on the pyrolyzed polymer matrix. The difference in the nano‐FTIR spectra indicated the chemical inhomogeneities within the pyrolyzed membrane. Similarly, the nano‐FTIR spectra in locations e–h, which were placed on the nanomaterials, exhibited variations in their chemical identities. In most cases (except locations a and f), the C–N and –CF_3_ footprints from the 6FDA structure were still maintained after pyrolysis, indicating the partial perseverance of the initial structure, which did not fully collapse. The variations of the peak positions, corresponding to C–N and –CF_3_, are clearly visualized in Figure 4g. More importantly, while the characteristic features of 6FDA‐DMN were identified, the characteristic features of pyrolyzed fullerenol (absorbance at ≈1410 cm^−1^) were not identified in any of the nano‐FTIR spectra. This indicated that the fullerenol particles were fully enveloped by the polymer matrix, although the agglomerated fullerenol exhibited a rather large size of ≈200–300 nm (Figure 4d). The significant difference in the comparison of the standard ATR‐FTIR and the average of the nano‐FTIR spectra can be observed in Figure 4h. In the average nano‐FTIR spectra of points a–d (located on the polymer matrix), the C–N peak was clearly identified; however, –CF_3_ seemed to be completely diminished. Similarly, the average nano‐FTIR spectra of points e–h (located on the nanomaterials) also showed the remaining C–N footprint and an almost unidentifiable –CF_3_ peak. However, in the nano‐FTIR spectra (Figure 4f), some locations still exhibited the presence of both C–N and –CF_3_ characteristic peaks. In contrast, C–N and –CF_3_ peaks were virtually unidentified in the spectra collected via standard ATR‐FTIR spectroscopy (Figure 4h). This comparison demonstrated the superiority of nano‐FTIR spectroscopy in revealing the chemical information at the local domain and unveiling chemical inhomogeneities within a material.

**Figure 5:**
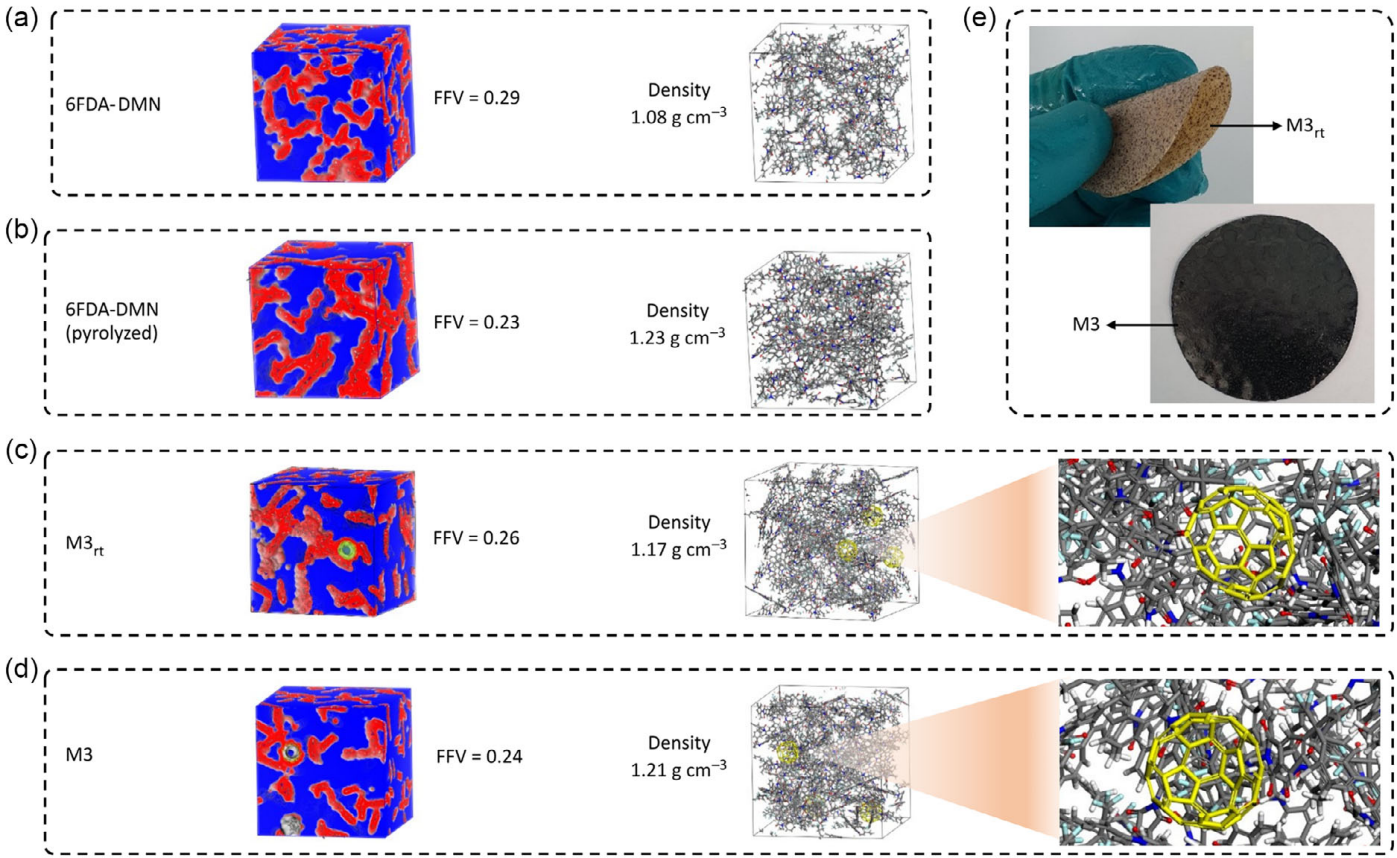
a–d) Fractional free volume of pristine 6FDA‐DMN (a), pyrolyzed 6FDA‐DMN (b), M3_rt_ (c), and M3 (d). e) Photograph of M3_rt_ and M3. The size of the simulation box (lattice) is 50 × 50 × 50 Å. The red color in the simulation box represents the volume of the polymer, and the blue color represents empty spaces.

Further, molecular dynamic simulation was conducted for 6FDA‐DMN and M3 before and after pyrolysis (M3 before pyrolysis named as M3_rt_) to investigate the polymer packing in the composite membranes. The photographs of M3_rt_ and its pyrolyzed counterpart (M3) are shown in **Figure** **5**e, where pyrolysis transformed the color of the composite membranes from brown to black. After the densities of the membranes were measured, the FFV values were calculated, and their models are visualized in Figure 5a–d. Although the final structures of the pyrolyzed membranes were unknown, in this simulation, we assumed the pyrolyzed membranes to have the same structure as that of the initial 6FDA‐DMN with higher density. The correlation between density and FFV is known to be inversely proportional, for example, if the density increased and then the FFV decreased. We experimentally measured the density of the membranes that we studied to estimate the relative FFV values for those membranes. Pyrolyzing the pristine 6FDA‐DMN increased its density from 1.08 to 1.23 g cm^−3^. Accordingly, their FFV values decreased from 0.29 to 0.23. The incorporation of fullerenols into the 6FDA‐DMN matrix resulted in a denser membrane (M3_rt_) with a density of 1.17 g m^−3^ (FFV = 0.26) compared to pristine 6FDA‐DMN. Pyrolyzing the composite 6FDA‐DMN/fullerenol membrane resulted in an ISA‐CCMS membrane (M3) with a compromised porosity, where the FFV (0.24) was higher than the FFV of the pyrolyzed polymer matrix (0.23) and lower than the FFV (0.26) of the composite 6FDA‐DMN/fullerenol (M3_rt_). Interestingly, 6FDA‐DMN lost ≈21% of its FFV after pyrolysis, whereas M3 lost only 7% of its FFV after pyrolysis. This indicates that the presence of fullerenols helps lower the chain packing after carbonization, which results in higher porosity in the membranes.

We hypothesize that pyrolysis induces membrane densification in all parts of the polymer arrangements. Meanwhile, intercalating fullerenol nanomaterials in between polymer chains followed by pyrolysis induces both shrinkage and enlargement in different parts of the polymer arrangements. This densification is manifested from the coordination‐induced rearrangement and the altered packing of the polymer chains, which is in line with the previously discussed N_2_ and CO_2_ sorption, as well as XRD analyses (Figure 2d–g). The molecular dynamic modeling suggests that the FFV of the membranes can be adjusted by combining nanomaterial intercalation and pyrolysis strategies.

### 2.2 Molecular Sieving Performance

**Figure 6:**
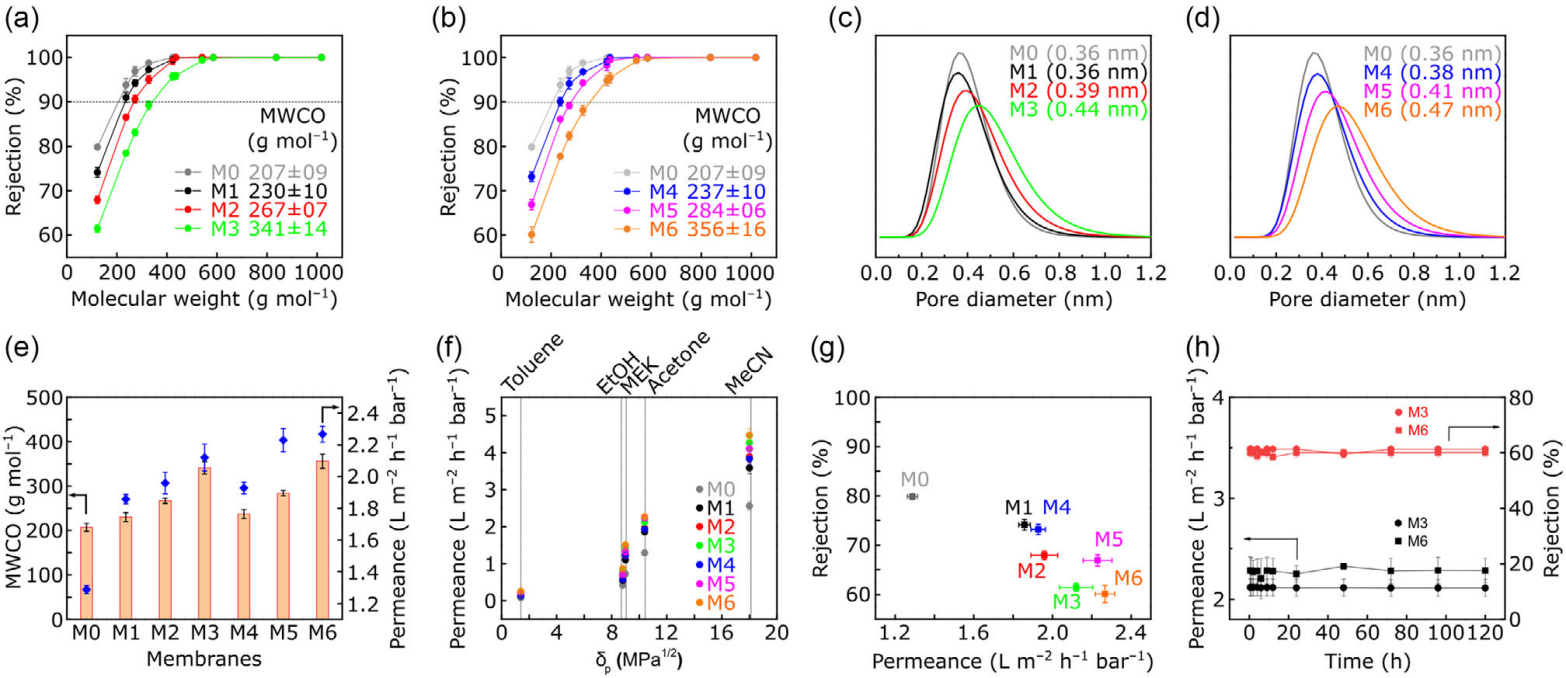
a,b) Rejection curves of M0–M3 (a) and M0–M6 (b); c,d) pore size distribution of M0–M3 (c) and M0–M6 (d); e) MWCO and permeance of M0–M6; f) correlation between solvent parameter and permeance in M0–M6; g) correlation between permeance and rejection in M0–M6; h) long‐term stability of M3 and M6. All the nanofiltration experiments were performed in acetone at 10 bar and 20 °C. For long‐term stability, 1‐phenylethanol was used as the solute.

The rejection profiles of the C_60_(OH)‐ and C_70_(OH)‐based ISA‐CCMS membranes, namely, M1–M3 and M4–M6, are shown in **Figure** **6**a,b, respectively. Compared with the M0 benchmark ISA‐CMS,^[^ ^7^ ^]^ all the ISA‐CCMS membranes exhibited higher molecular weight cutoff (MWCO) values. Notably, the increase in fullerenol contents in the ISA‐CCMS membranes was linearly correlated with the increase in the MWCO values. For instance, a 10% increase due to the MWCO of M0 was observed after incorporating 1 wt% of C_60_(OH) in the fabrication of M1, corresponding to a change from 207 to 230 g mol^−1^. Meanwhile, incorporating 2 wt% of C_60_(OH) in the ISA‐CCMS membrane (M2) resulted in the shift of the MWCO value to 267 g mol^−1^, which was 14% higher than that of M1. A further increase in C_60_(OH) content to 3 wt% (M3) shifted the MWCO value by 22% from 267 to 341 g mol^−1^. A similar trend was also observed when incorporating various contents of C_70_(OH) into ISA‐CCMS membranes. For example, incorporating 1, 2, and 3 wt% of C_70_(OH) resulted in membranes with MWCO values of 237, 284, and 356 g mol^−1^, respectively. Interestingly, for each content level of nanomaterials, ISA‐CCMS membranes intercalated with C_70_(OH) exhibited higher MWCO values than those of ISA‐CCMS membranes containing C_60_(OH). The rejection results were in line with the surface area measurements (Figure 2d), where the large size and elongated shape of C_70_(OH)‐induced larger ISA‐CCMS membrane porosities than those created using C_60_(OH). These results indicate that the type and concentration of intercalated nanomaterials strongly affected the molecular sieving performance. The obtained MWCO values for all the CCMS membranes were within the range of the tightest membranes reported for OSN (Table S9, Supporting Information).

Indeed, the linearity between the fullerenol contents and the MWCO values was also reflected by the pore size of the membranes, as shown in Figure 6c,d. The enlargement in the pore diameter due to the increase in the fullerenol content was induced by the fullerenol intercalation in between the polymer chains. Pore diameter analysis revealed the importance of the type and concentration of the intercalated nanomaterials in controlling the pore diameter of the membranes. The pore size distributions obtained from the nanofiltration measurement (between 0.3 and 0.5 nm) were the smaller than those determined from the CO_2_ sorption measurement (between 0.5 and 0.9 nm). These discrepancies originate from at least two reasons. The first reason could be related to smaller size of CO_2_ compared to solute molecules, thus enabling probing a larger area of CCMS and accessing the majority of the pores that solute molecules cannot access. The second reason could be due to measuring a different form of the material: in nanofiltration, the pore size was determined from the flat sheet membrane which generally exhibited smaller surface area and pore sizes as compared to the powder form when measured in a CO_2_ sorption experiment.

As observed in Figure 6a,b, the increment in the MWCO was only around 125 g mol^−1^ (from M1 to M6), which translated to the increment in the pore size of around 0.1 nm (Figure 6c,d). Such a small increment could not be resulted from the effect of large agglomerates, but rather a smaller filler size. Therefore, we believe that some fullerenols are dispersed in a much smaller size than those agglomerates observed from the surface SEM images.

The solvent permeance (Figure 6e) exhibited the same trend as that of the membrane MWCO and pore diameter. The increase in the fullerenol content enhanced the membrane permeance because of the gradual enlargement of the pore size. M1, M2, and M3 showed acetone permeances of 1.86, 1.96, and 2.12 L m^−1^ h^−1^ bar^−1^, respectively. A 4% increase (from 1.86 to 1.93 L m^−1^ h^−1^ bar^−1^ for M1 and M4, respectively) in acetone permeance was observed when replacing 1 wt% of C_60_(OH) in M1 with 1 wt% of C_70_(OH) in M4 as the filler. The acetone permeance improved by 12% when 2 wt% of C_60_(OH) in M2 was replaced with 2 wt% of C_70_(OH) in M5 as the filler. A 3 wt% C_70_(OH) incorporation in the ISA‐CCMS membrane (M6) led to an acetone permeance of 2.27 L m^−1^ h^−1^ bar^−1^, which corresponded to a 7% increase compared to M5. Notably, a 40% higher permeance was achieved from ISA‐CCMS membranes prepared with the highest fullerenol loading (M3 and M6) compared with that of the pristine ISA‐CMS (M0).

We further examined the permeation of five solvents with different polarities to investigate the effect of fullerenol intercalation on molecular sieving (Figure 6f). The solvent permeance was found to be directly correlated to the solvent polarity. An increased solvent polarity resulted in higher solvent permeance. The acetonitrile solvent, which had the highest polarity factor (*δ*_p_), demonstrated the highest permeance, ranging from 3.5 to 4.5 L m^−2^ h^−1^ bar^−1^ for M1–M6. Meanwhile, the least polar solvent (i.e., toluene) exhibited the lowest permeance (from 0.1 to 0.2 L m^−2^ h^−1^ bar^−1^) among all the tested membranes. These phenomena could have resulted from the increased affinity between less polar solvents and the carbon matrix, which led to the lower permeance of less polar solvents and the higher permeance of more polar ones. Additionally, we also investigated the correlation between solvents’ viscosity and molecular diameter with their permeance (Figure S8, Supporting Information). There is no clear linear correlation between the solvent permeance with solvent viscosity and molar diameter, which showed that the polarity effect dominated the solvent permeance through the membrane.

Figure 6g compares the performance of all the ISA‐CCMS membranes. The trade‐off between solute rejection and solvent permeance can be fine‐tuned by controlling the type and amount of the fullerenol nanomaterial. For instance, under the same content values, C_70_(OH) displayed slightly higher permeance and lower rejection than C_60_(OH). Further, the increase in the content of the fullerenol nanomaterials in the ISA‐CCMS membranes decreased the rejection but increased the solvent permeance.

Furthermore, the **M3** and M6 membranes were evaluated for their long‐term stability and exhibited robust performance over 120 h of continuous crossflow filtration in acetone at 10 bar (Figure 6h). M3 and M6 displayed a stable permeance at ≈2.1 and 2.2 L m^−2^ h^−1^ bar^−1^, respectively. In addition, stable 61.1% and 59.8% rejections of 1‐phenylethanol (molecular weight of 122.17 g mol^−1^) were demonstrated by M3 and M6, respectively.

Our results suggest that ISA‐CCMS membranes are excellent candidates for liquid separation applications. The molecular sieving performance demonstrated that these membranes fall within the category of tight membranes that are suitable for OSN and OSRO applications.

## 3 Conclusion

ISA‐CCMS membranes were developed from intrinsically microporous 6FDA‐DMN polyimide intercalated with C_60_(OH) and C_70_(OH) fullerenols. The chemical footprints of the pyrolyzed membranes and their chemical inhomogeneity at the nanodomain level were revealed via nano‐FTIR spectroscopy. The variability in the peak positions of nano‐FTIR spectra corresponding to C–N and –CF_3_ was identified, indicating anisotropy in the chemistry of the membrane induced by pyrolysis. Furthermore, fullerenol nanomaterials were found to be fully enveloped by the polymer matrix, indicated by the absence of the nano‐FTIR spectra characteristic of pyrolyzed fullerenol (≈1410 cm^−1^). Further, the incorporation of fullerenol nanomaterials altered the distance between the polymer chains in different parts.

The changes in the FFV were also investigated by performing molecular dynamic simulations. The synergistic effects of pyrolysis and fullerenol incorporation resulted in ISA‐CCMS membranes with controllable porosity. The footprints of the size and molecular shape of the fullerenols translated from material properties to molecular sieving performance. We found that the morphology (size and geometrical shape) of the nanomaterials played a more significant role in enhancing the ISA‐CCMS performance compared to the porosity of the nanomaterials. Furthermore, the free‐standing ISA‐CCMS membranes demonstrated excellent potential for solute separation in various organic media. The size‐sieving properties of the ISA‐CCMS membranes were fine‐tuned by adjusting the type and amount of fullerenols.

This research demonstrates the rational design to revolutionize the strategy in fabricating ISA‐CCMS material systems and provides a comprehensive study on ISA‐CCMS membrane characteristics from nanodomain‐ and molecular‐level analysis to the structure–property–performance nexus. The rational design demonstrated herein considered five essential pillars: 1) the geometrical aspects of nanomaterials, 2) nanomaterial dispersibility, 3) heat‐induced phase transformation of nanomaterials, 4) encapsulation of nanomaterials by polymer matrices, and 5) nanodomain footprinting and local chemical analysis of the ISA‐CCMS membrane.

## 4 Experimental Section

4.1

4.1.1

##### Materials

Chemicals and solvents used in the polymer synthesis were as follows: 6FDA (99%), DMN, isoquinoline (97%), *m*‐cresol (99%), chloroform (≥99.5%), *N*‐methyl‐2‐pyrrolidone (NMP, 99.9%), and methanol (≥99.9%).

Solvents used for membrane stability test were as follows: tetrahydrofuran (≥99.9%), dimethylformamide (≥99.9%), dimethyl sulfoxide (≥99.7%), dimethyl acetamide (≥99.7%), chloroform (≥99.5%), NMP (99.9%), dichloromethane (≥99.8%), acetone (98%), *m*‐cresol (99%), hydrochloric acid (37%), trifluoroacetic acid (99%), acetic acid (≥99.7%), sulfuric acid (95.0%–98.0%), nitric acid (70%), propionic acid (≥99.5%), acetic anhydride (≥99%), sodium hydroxide, potassium hydroxide, potassium tert‐butoxide, trimethylamine (≥99.5%), and ammonium hydroxide (28%–30%). All solvents and chemicals were of high‐performance liquid chromatographic grade and used as received unless stated otherwise.

Chemicals and solvents used as solutes in nanofiltration experiment: 1‐phenylethanol (98%), styrene dimer (97%), estradiol (98%), oleuropein (98%), valsartan (98%), rose bengal (95%), methyl orange (85%), losartan (95%), acid fuchsine, ethanol (≥99.8%), methethylketone (≥99.5%), acetonitrile (≥99.9%), acetone (≥99.8%), toluene (≥99.5%).

##### Polymer Synthesis

The 6FDA‐DMN polyimide was prepared following a previously reported procedure^[^ ^7^ ^]^ via a polycondensation reaction of an equimolar amount of 6FDA and DMN at 200 °C (Scheme S1, Supporting Information). Monomers and *m*‐cresol were added to a two‐neck round‐bottom flask equipped with a nitrogen inlet. Then, the reaction mixture was stirred at 100 °C for 0.5 h; thereafter, 0.1 mL of isoquinoline was added. After 20 min, the reaction temperature was gradually increased to 200 °C and then maintained at the same temperature for 30 min to ensure full imidization. Thereafter, the viscous solution was poured into 500 mL of methanol and stirred for 4 h, and the resulting product was collected through filtration. For further purification, the polymer solution was reprecipitated in methanol thrice. The final polymer fibers were dried in a vacuum oven at 150 °C for 24 h, and the molecular structures were confirmed through ^1^H NMR, ^19^F NMR, and FTIR spectroscopy. 6FDA‐DMN (6.0 g, yield: 95%). ^1^H NMR (400 MHz, CDCl_3_, δ): 2.47 (s, 6 H), 7.35 (br s, 2 H), 7.49–7.58 (m, 6 H), 7.67 (m, 2 H), 8.08–8.11 (m, 4 H), and 8.19–8.22 (m, 2 H). 19 F NMR (376 MHz, CDCl_3_, δ): 63.0. Number average molecular weight (*M*_n_) = 68,000 g mol^−1^, molecular weight (*M*_w_) = 84,500 g mol^−1^, and polydispersity index (PDI) = 1.24.

##### ISA‐CCMS Membrane Fabrication

First, 6FDA‐DMN was dissolved in NMP at 15 wt% dope solution concentration. Then, the obtained solution was stirred at room temperature (22 °C) for 12 h, followed by adding fullerenols at different concentrations (i.e., 1, 2, and 3 wt%). The membranes derived from 1, 2, and 3  wt% C_60_(OH) nanomaterials in the dope solution were designated M1, M2, and M3, respectively. Similarly, the membranes derived from 1, 2, and 3 wt% C_70_(OH) nanomaterials in the dope solution were designated M4, M5, and M6, respectively. The benchmark membrane without fullerenol was designated as M0 and fully characterized in our previous publication.^[^ ^7^ ^]^ The composite solution was stirred for 24 h to obtain a homogenous solution and then placed in an incubator shaker (IKA KS 4000) for 24 h for complete dissolution and gas bubble removal. The homogeneous solution was cast on the top of a glass plate using an Elcometer4340 automatic film applicator using a blade with a thickness of 250 μm and transverse speed of 150 m h^−1^. The obtained films were phase inverted (within 3–5 s) via immersion into a coagulation bath containing deionized water with a resistivity of 18.2 MΩ cm (Type II) collected using a Milli‐Q system. The water in the coagulation bath was changed three times to ensure the total removal of NMP. The room temperature and relative humidity were 22 °C and 58%, respectively. Then, the membrane coupons with 5 cm diameters were placed between two stainless steel sheets and inserted into a Carbolite furnace equipped with a nitrogen inlet. The membranes were heated at 600 °C for 1 h at a ramp rate of 2 °C min^−1^. Then, the furnace was cooled down to room temperature, and the carbon membranes were collected and used for testing.

##### Polymer and Membrane Characterizations

The chemical structure of 6FDA‐DMN was characterized through ^1^H and ^19^F NMR using a Bruker AVANCE‐III spectrometer (400 MHz), and FTIR spectroscopy, using a Thermo Fisher Scientific Nicolet spectrometer (iS10) equipped with diamond‐ATR imaging, was conducted to collect the FTIR spectra at wavenumbers of 600–4000 cm^−1^. Nano‐FTIR spectroscopy (Neaspec GmbH, Germany) was performed by applying laser centering at a wavelength of ≈1333 cm^−1^. A Pt/Ir‐coated AFM tip with a frequency of 75 kHz was used. The membrane sample was adhered onto a silicon wafer by taping the edges of the membrane using silver tape, and silicon wafer reflection was used to calibrate the instrument. An area of 1.5 × 1.5 μm was selected for the collection of the nano‐FTIR spectra. The thermal degradation of 6FDA‐DMN was examined via thermogravimetric analysis (Q5000, TA Instruments, USA) at up to 800 °C in a nitrogen atmosphere. The structural characterizations of the ISA‐CCMS membranes (M1–M6) were evaluated using wide‐angle XRD performed on a Bruker D8 Advance diffractometer; the *d*‐spacing between adjacent polymer chains were estimated from the WDXRD peak using Bragg's law (*n*λ = 2*d*sin*θ*). The mechanical properties of all membranes were tested by nanoindentation using a NanoTest Vantage instrument. The membrane samples were adhered to a silicon wafer before the experiment, and the sample‐loaded wafer was placed in the holder of the NanoTest Vantage instrument. The obtained mechanical properties are reported as the average of three measurements. The BET surface area and pore size distribution obtained through nonlocal density‐functional theory were analyzed using the nitrogen adsorption isotherms, which were obtained using the Micromeritics ASAP 2020 analyzer at −196 °C. The membrane samples were ground to become powder before the measurements and dried for 12 h at 150 °C using ASAP degassing ports. The nitrogen adsorption isotherms were recorded at up to 1 bar, whereas the CO_2_ adsorption isotherms were recorded using ASAP 2050 instrument at 0 °C and up to 1 bar. The images of the membrane surfaces and cross sections were acquired using a Novanano instrument at 5 kV high voltage and 24 pA current. EDX analysis was conducted on a Teneo VS (FEI, USA) SEM at 20 keV to identify the elemental compositions in the fullerenol nanomaterials and polymer membrane matrix. The particle size distributions of the fullerenols on the membranes were measured using the ImageJ software. The surface and roughness of the membranes were evaluated via AFM (Agilent 5500). The water contact angles (WCAs) of the CCMS membranes were measured using a contact angle goniometer (Krüss Scientific, Germany). The sessile drop method was employed, and the Young–Laplace fitting was used. The average WCA value was obtained from three measurements. Molecular modeling and the FFV of the membranes were determined using BIOVIA Materials Studio 2017 after measuring the density of each membrane. The densities of the membranes were determined via Archimedes’ principle using the liquid saturation method in isooctane (ρ_25 °C_ = 0.68 g mL^−1^). The lattice parameter for all cubic amorphous cells presented by the model was set to 50 Å, the ratio between the polymer and fullerenol was set to 97:3 (wt%:wt%), and the molecular dynamic simulation was performed for standard ambient temperature and pressure (25 °C at 1 atm). The COMPASS II force field was applied to assign force field parameters to the simulations.

##### Molecular Sieving Performance

Prior to nanofiltration tests, the membrane stabilities in various conditions (organic solvents, acids, and bases) were evaluated by observing if the membrane was soluble or not after immersing in the respective solvents, acids, and bases for 24 h (Table S2–S4, Supporting Information). The separation performance of the membranes was determined using a crossflow nanofiltration apparatus. Ten solute molecules as listed in the materials section were used in the nanofiltration test to determine the rejection profiles of the studied membranes. The concentration polarization at the membrane surface was reduced using a recirculation pump (Michael Smith Engineers, UK), which also ensured a homogeneous solute concentration in the retentate loop. The retentate was recirculated at a rate of 1.2 L min^−1^. All membranes were rinsed with and soaked in acetonitrile and then conditioned at an applied pressure of 10 bar for 16 h. At equilibrium, the solvent flux, permeance, and solute rejection values were measured using Equation ((1), (2))–(3). The active area of the membrane coupon was 25 cm^2^. The solute rejection values were determined from the ratio of the permeate and retentate concentrations of the solutes. Standard polystyrene markers containing 1 g L^−1^ PS580 and PS1300 with 0.1 g L^−1^ styrene dimer (236 g mol^−1^) were used for nanofiltration to obtain the MWCO curves^[^ ^18^ ^]^. All other solute concentrations in the feed were 10 μM.

(1) $$\text{Flux }\left[{\text{L m}}^{2}h^{-1}\right]=\frac{V}{A\times t}$$

(2) $$\text{Permeance }\left[{\text{L m}}^{2}h^{-1}{\text{bar}}^{-1}\right]=\frac{V}{\Delta P\times A\times t}$$

(3) $$\text{Rejection}\left[\%\right]=\left(1-\frac{C_{p}}{C_{r}}\right)\times 100$$

where *V* is the volume of the solvent permeating through a given membrane area (*A*) at a given time (*t*), Δ*P* is the transmembrane pressure, and *C*_p_ and *C*_r_ are the solute concentrations of the permeate and retentate streams, respectively. The MWCO value was estimated based on the styrene rejection profiles at 90% rejection from two separate runs using different membrane coupons.

##### Pore Size Calculation

The pore sizes of the membranes were determined from the predicted rejection curves which were developed using neutral molecules as solutes, such as polystyrenes with various molecular weights. The molecular size of those polystyrenes can be correlated with their diffusivity and molecular size (solute molar volume and Stokes radius) by using Wilke–Chang (Equation (S11)) and Stokes–Einstein equations (Equation (S10)). The detailed mathematical derivations are shown in Section 3. Pore size calculation, in the Supporting Information. The molecular weight at 90% rejection was then associated to determine the pore size of the membrane. Form this predicted rejection curve, the actual dye molecules (with various molecular weight) were fitted and their corresponding molecular weight at 90% rejection can be correlated to the pore size of the membrane.

## Conflict of Interest

The authors declare no conflict of interest.

## Supporting information

Supplementary Material

## Data Availability

The data that support the findings of this study are available in the supplementary material of this article.
