# Supplementary material for: Nanodomain Control in Carbon Molecular Sieve Membranes via Nanomaterial Footprinting
Source: Small Sci. 2023 Dec 3;4(1):2300162. doi: 10.1002/smsc.202300162 (PMC11935167; doi:10.1002/smsc.202300162)
Supplement: Supplementary file 1 — Supplementary Material [file SMSC-4-2300162-s001.pdf]

Supporting Information

Nanodomain control in carbon molecular sieve membranes via nanomaterial footprinting

*Rifan Hardian[1#], Mahmoud. A. Abdulhamid[1#], Gyorgy Szekely[1,2]**

[1]Advanced Membranes and Porous Materials Center, Physical Science and Engineering Division (PSE), King Abdullah University of Science and Technology (KAUST), Thuwal, 23955-6900, Saudi Arabia

[2]Chemical Engineering Program, Physical Science and Engineering Division (PSE), King Abdullah University of Science and Technology (KAUST), Thuwal, 23955-6900, Saudi Arabia

*gyorgy.szekely@kaust.edu.sa, www.szekelygroup.com, +966128082769

[#]The authors equally contributed to this work

Table of contents

1. **Characterizations** 3
2. **Nanofiltration performance** 8
3. **Pore-size calculation** 12
4. **References** 14

List of figures

Figure S1. ^{1}H NMR spectrum of 6FDA-DMN in $CDCl_3$ 3
Figure S2. ^{19}F NMR spectrum of 6FDA-DMN in $CDCl_3$. 4
Figure S3. FTIR spectrum of 6FDA-DMN polyimide. 4
Figure S4. EDX spectra of $C_{60}(OH)$ 5
Figure S5. Particle-size analysis using the ImageJ software. 5
Figure S6. Cross-section SEM images of CCMS membranes. 6
Figure S7. (a) CO_2 adsorption isotherms at 0 ℃ and (b) pore size distribution calculated using CO_2 – DFT model. 6
Figure S8. Correlation between solvent's viscosity and permeance (a), and between solvent's molecular diameter and permeance. 11
Figure S9. Schematic of the multistage crossflow nanofiltration apparatus used for membrane 11

List of tables

Table S1. BET surface area, water contact angle, and hardness values of CCMS membranes. 7
Table S2. CCMS membrane stability in organic solvents. √=soluble; ✗=insoluble. 7
Table S3. CCMS membrane stability in acids. √=soluble; ✗=insoluble. 7
Table S4. CCMS membrane stability in bases [1 M]. √=soluble; ✗=insoluble. 7
Table S5. Rejection data of various solutes in M0–M6. 8
Table S6. MWCO values of the membranes and their acetone permeances. 8
Table S7. Solvent polarities and their permeances through M0–M6. 9
Table S8. Long-term stability of M3 and M6 over a continuous OSN at 10 bar and 20°C. Acetone was used as the solvent, and 1-phenylethanol was used as the solute. 9
Table S9. Permeance and MWCO of the CCMS membranes compared to tightest OSN membranes 10

1. Characterizations

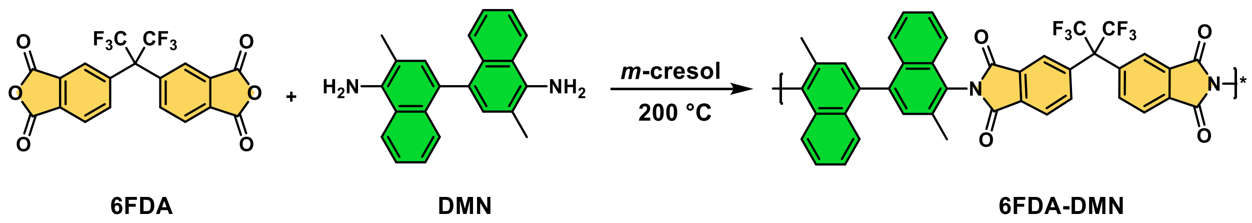

Scheme S1. Synthesis of intrinsically microporous 6FDA-DMN polyimide.[1]

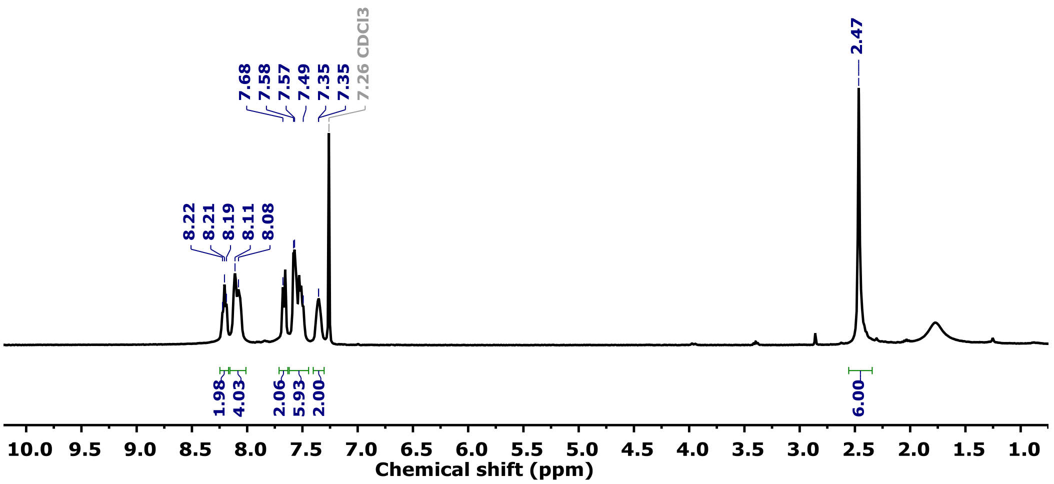

Figure S1. ^{1}H NMR spectrum of 6FDA-DMN in $CDCl_3$.

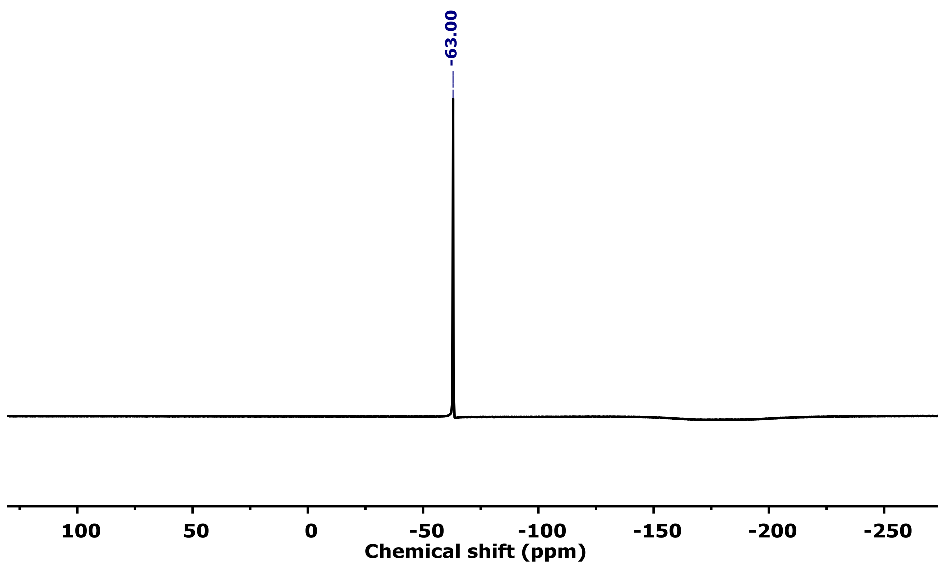

Figure S2. ^{19}F NMR spectrum of 6FDA-DMN in $CDCl_3$.

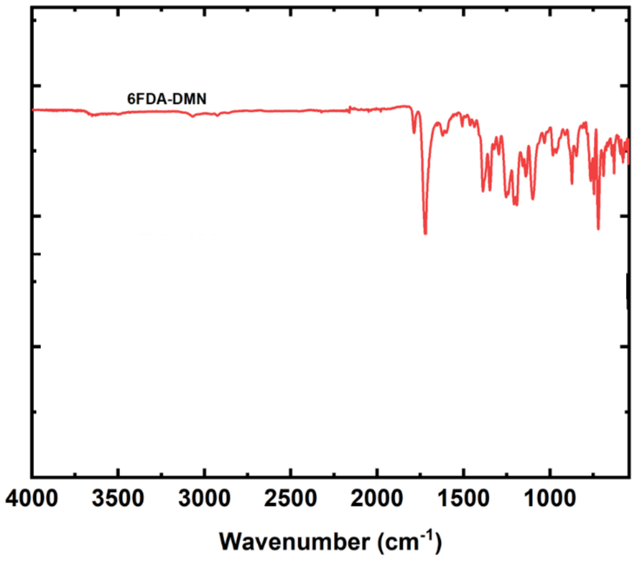

Figure S3. FTIR spectrum of 6FDA-DMN polyimide.

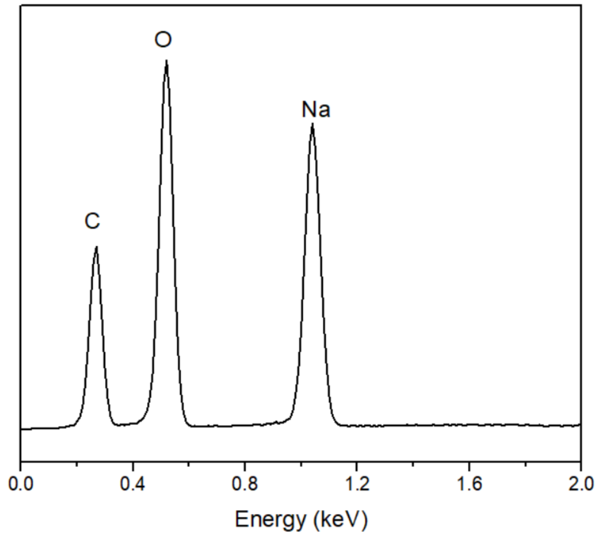

Figure S4. EDX spectra of $C_{60}(OH)$.

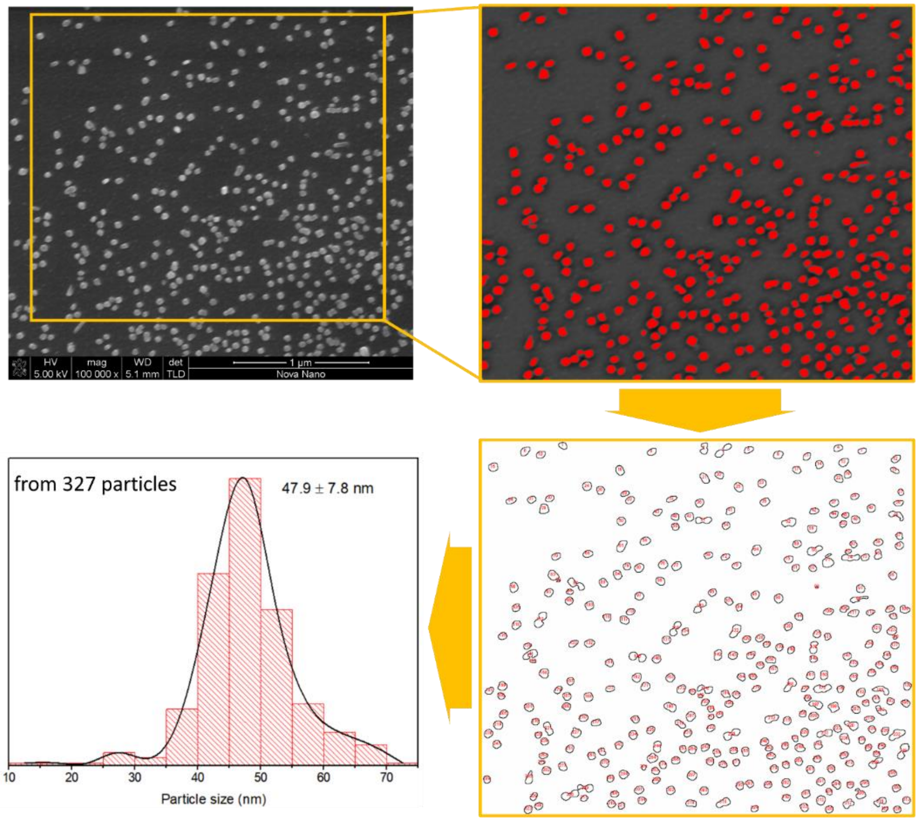

Figure S5. Particle-size analysis using the ImageJ software.

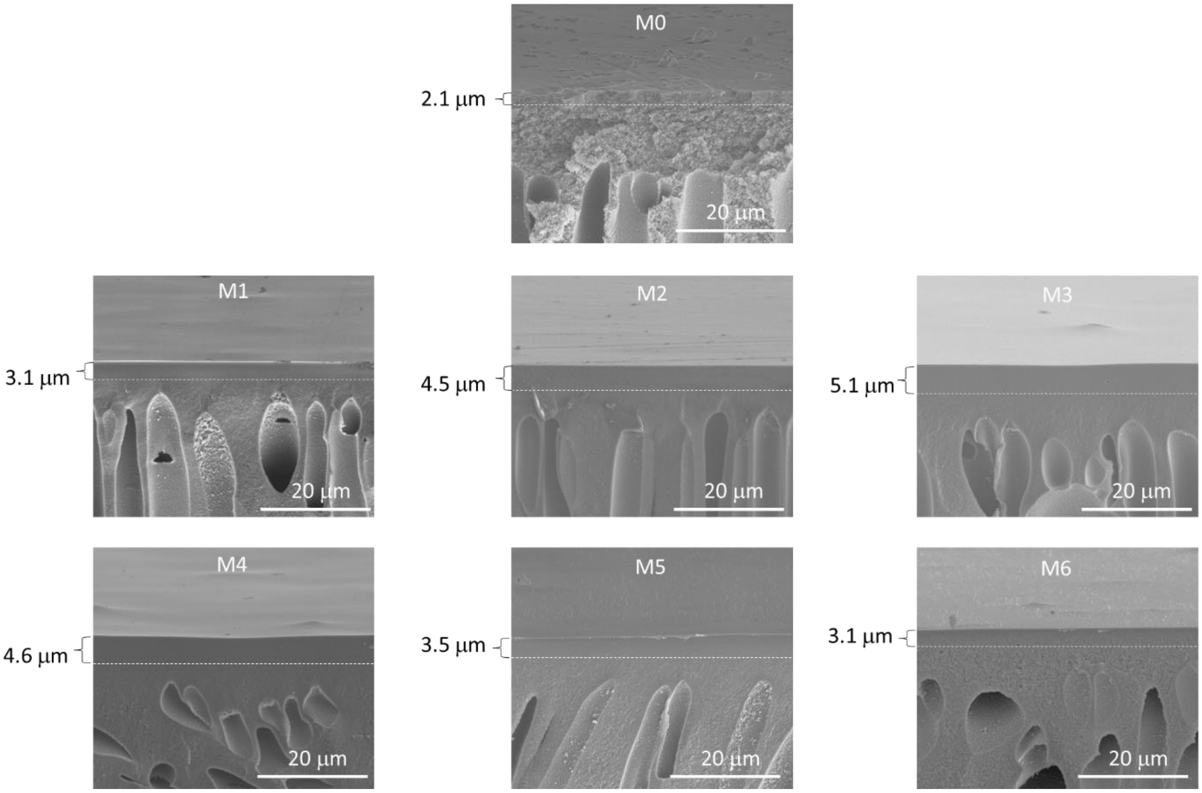

Figure S6. Cross-section SEM images of CCMS membranes.

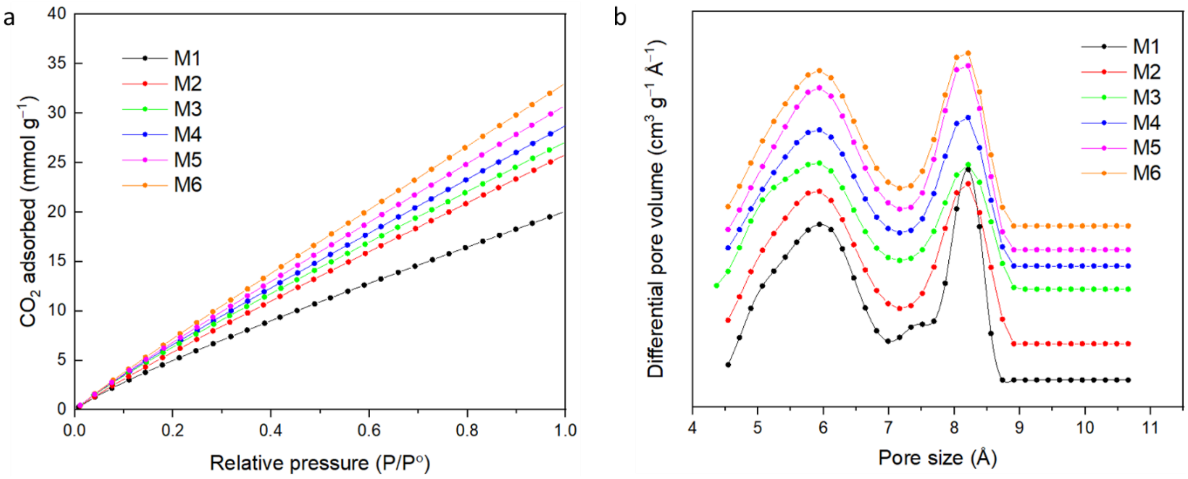

Figure S7. (a) CO_2 adsorption isotherms at 0 °C and (b) pore size distribution calculated using CO_2 – DFT model.

Table S1. BET surface area, water contact angle, and hardness values of CCMS membranes.

	BET ($m^2\ g^{-1}$)		WCA (°)	Hardness (GPa)
	N_2	CO_2		
M1	628.8 ± 17.8	710.5 ± 12.4	79.2 ± 2.3	0.063 ± 0.003
M2	641.4 ± 13.1	921.4 ± 17.8	76.2 ± 3.4	0.042 ± 0.010
M3	651.9 ± 12.9	940.2 ± 20.4	82.1 ± 3.5	0.053 ± 0.009
M4	652.5 ± 13.1	997.2 ± 22.3	77.4 ± 4.1	0.058 ± 0.021
M5	674.8 ± 13.3	1101.5 ± 21.9	78.5 ± 2.7	0.072 ± 0.027
M6	690.1 ± 12.5	1158.2 ± 28.9	81.3 ± 3.3	0.061 ± 0.014

Table S2. CCMS membrane stability in organic solvents. √=soluble; ✗=insoluble.

Membrane	THF	DMF	DMSO	DMAc	NMP	Acetone	m-cresol	DCM	Chloroform
M1	✗	✗	✗	✗	✗	✗	✗	✗	✗
M2	✗	✗	✗	✗	✗	✗	✗	✗	✗
M3	✗	✗	✗	✗	✗	✗	✗	✗	✗
M4	✗	✗	✗	✗	✗	✗	✗	✗	✗
M5	✗	✗	✗	✗	✗	✗	✗	✗	✗
M6	✗	✗	✗	✗	✗	✗	✗	✗	✗

Table S3. CCMS membrane stability in acids. √=soluble; ✗=insoluble.

Membrane	Trifluoroacetic acid	Acetic acid	HCl	H_2SO_4	HNO_3	Propionic acid	Acetic anhydride
M1	✗	✗	✗	✗	✗	✗	✗
M2	✗	✗	✗	✗	✗	✗	✗
M3	✗	✗	✗	✗	✗	✗	✗
M4	✗	✗	✗	✗	✗	✗	✗
M5	✗	✗	✗	✗	✗	✗	✗
M6	✗	✗	✗	✗	✗	✗	✗

Table S4. CCMS membrane stability in bases [1 M]. √=soluble; ✗=insoluble.

Membrane	NaOH	KOH	KOtBu	TEA	Ammonium hydroxide
M1	✗	✗	✗	✗	✗
M2	✗	✗	✗	✗	✗
M3	✗	✗	✗	✗	✗
M4	✗	✗	✗	✗	✗
M5	✗	✗	✗	✗	✗
M6	✗	✗	✗	✗	✗

2. Nanofiltration performance

Table S5. Rejection data of various solutes in M0–M6.

Solutes	Molecular weight (g mol^{-1})	Rejection (%)						
		M0	M1	M2	M3	M4	M5	M6
1-Phenylethanol (PHE)	122.17	79.85 ± 0.55	74.13 ± 1.09	67.94 ± 0.87	61.45 ± 0.84	73.17 ± 1.06	66.88 ± 1.16	60.13 ± 1.76
Styrene dimer (SD)	236.4	93.83 ± 1.45	90.97 ± 1.08	86.54 ± 0.62	78.48 ± 0.57	90.09 ± 0.94	86.10 ± 0.29	77.76 ± 0.27
Estradiol (ED)	272.38	96.89 ± 1.04	94.24 ± 0.77	90.63 ± 0.90	83.15 ± 0.79	94.12 ± 1.25	89.19 ± 0.73	82.40 ± 0.92
Methyl orange (MO)	327.33	98.76 ± 0.35	97.27 ± 0.45	95.02 ± 0.89	89.21 ± 0.96	96.75 ± 0.45	94.29 ± 0.32	88.10 ± 1.12
Losartan (LS)	422.92	100.00 ± 0.00	99.45 ± 0.55	99.22 ± 0.78	95.76 ± 0.83	99.11 ± 0.63	98.22 ± 1.13	94.81 ± 1.14
Valsartan (VS)	435.52	100.00 ± 0.00	100.00 ± 0.00	100.00 ± 0.00	95.85 ± 0.78	100.00 ± 0.00	99.48 ± 0.52	95.39 ± 1.12
Oleuropein (OR)	540.51	100.00 ± 0.00	100.00 ± 0.00	100.00 ± 0.00	99.39 ± 0.61	100.00 ± 0.00	100.00 ± 0.00	99.31 ± 0.52
Acid fuchsin (AF)	585.54	100.00 ± 0.00	100.00 ± 0.00	100.00 ± 0.00	100.00 ± 0.00	100.00 ± 0.00	100.00 ± 0.00	99.81 ± 0.19
Roxithromycin (RT)	837.05	100.00 ± 0.00	100.00 ± 0.00	100.00 ± 0.00	100.00 ± 0.00	100.00 ± 0.00	100.00 ± 0.00	100.00 ± 0.00
Rose bengal (RB)	1017.65	100.00 ± 0.00	100.00 ± 0.00	100.00 ± 0.00	100.00 ± 0.00	100.00 ± 0.00	100.00 ± 0.00	100.00 ± 0.00

Table S6. MWCO values of the membranes and their acetone permeances.

Membranes	MWCO (g mol^{-1})	Standard deviation	Permeance (L m^{-2} h^{-1} bar^{-1})	Standard deviation
M0	207	9	1.28	0.02
M1	230	10	1.85	0.02
M2	267	6	1.95	0.06
M3	341	14	2.12	0.08
M4	237	10	1.92	0.03
M5	284	6	2.22	0.07
M6	356	16	2.26	0.04

Table S7. Solvent polarities and their permeances through M0–M6.

Solvents	Polarity ($MPa^{1/2}$)	Permeance ($L\ m^{-2}\ h^{-1}\ bar^{-1}$)						
		M0	M1	M2	M3	M4	M5	M6
Acetonitrile	18.00	2.56 ± 0.13	3.58 ± 0.16	3.89 ± 0.20	4.27 ± 0.20	3.83 ± 0.14	4.10 ± 0.18	4.47 ± 0.18
Acetone	10.40	1.29 ± 0.05	1.85 ± 0.03	1.95 ± 0.07	2.12 ± 0.08	1.92 ± 0.04	2.22 ± 0.07	2.26 ± 0.05
MEK	9.00	0.72 ± 0.08	1.10 ± 0.10	1.24 ± 0.08	1.42 ± 0.1	1.21 ± 0.09	1.32 ± 0.11	1.49 ± 0.12
EtOH	8.80	0.42 ± 0.04	0.54 ± 0.06	0.68 ± 0.07	0.78 ± 0.09	0.57 ± 0.04	0.71 ± 0.08	0.85 ± 0.10
Toluene	1.40	0.08 ± 0.01	0.12 ± 0.01	0.19 ± 0.04	0.24 ± 0.07	0.13 ± 0.02	0.20 ± 0.02	0.25 ± 0.02

Table S8. Long-term stability of M3 and M6 over a continuous OSN at 10 bar and 20°C. Acetone was used as the solvent, and 1-phenylethanol was used as the solute.

Time (h)	M3				M6			
	Rejection (%)	Standard deviation	Permeance ($L\ m^{-2}\ h^{-1}\ bar^{-1}$)	Standard deviation	Rejection (%)	Standard deviation	Permeance ($L\ m^{-2}\ h^{-1}\ bar^{-1}$)	Standard deviation
0.5	61.35	0.84	2.12	0.09	60.15	1.37	2.28	0.14
1	61.35	0.87	2.12	0.08	60.07	1.48	2.28	0.13
2	60.35	1.12	2.12	0.09	60.06	1.42	2.27	0.12
4	61.36	0.84	2.12	0.08	58.80	1.48	2.28	0.12
6	60.33	0.86	2.12	0.08	60.14	1.38	2.20	0.20
9	61.37	0.86	2.12	0.08	59.97	1.56	2.28	0.13
12	61.34	0.85	2.12	0.08	58.51	1.35	2.28	0.13
24	61.36	0.84	2.11	0.08	60.07	1.55	2.25	0.08
48	59.47	1.15	2.12	0.08	60.04	1.54	2.33	0.03
72	61.35	0.87	2.11	0.08	60.04	1.50	2.28	0.13
96	61.34	0.87	2.11	0.08	60.09	1.46	2.28	0.13
120	61.35	0.84	2.11	0.08	60.15	1.44	2.28	0.12

Table S9. Permeance and MWCO of the CCMS membranes compared to tightest OSN membranes

Membrane	Solvent	Permeance ($L\ m^{-2}\ h^{-1}\ bar^{-1}$)	MWCO ($g\ mol^{-1}$)	Ref
**M1**	Acetone	1.85	230	This work
**M2**	Acetone	1.95	267	This work
**M3**	Acetone	2.12	341	This work
**M4**	Acetone	1.92	237	This work
**M5**	Acetone	2.22	284	This work
**M6**	Acetone	2.26	356	This work
**CMS (15%, 250 μm, 500 °C)**	Acetone	1.733	291	1
**CMS (15%, 250 μm, 600 °C)**	Acetone	1.29	207	1
**CMS (15%, 200 μm, 600 °C)**	Acetone	1.585	209	1
**CMS (15%, 150 μm, 600 °C)**	Acetone	1.7945	208	1
**CMS (17%, 250 μm, 600 °C)**	Acetone	0.9395	188	1
**CMS (19%, 250 μm, 600 °C)**	Acetone	0.4545	137	1
**CMS_d (15%, 250 μm, 600 °C)**	Acetone	0.1265	<122	1
**M3: 24 wt% PI**	Toulene	1.66	250	3
**M4: 26 wt% PI**	Toulene	1.0	250	3
**M1 (DMF-dioxane-1:3)**	Toulene	3.6	<200	4
**M2 (DMF-dioxane-1:1)**	Toulene	-	420	4
**M3 (20 wt%)**	DMF	0.56	250	5
**M4 (18 wt%)**	DMF	0.6–0.9	420	5
**StarMem 122**	Toluene	1.16	220	6
**StarMem240**	Toulene	2.05	400	6
**PBI-H2SO4**	Methanol	3.5	500	7
**X-PBI**	IPA	5.8	440	8
**SPEEK (10 wt%)**	THF	0.42–0.52	395-495	9
**SPEEK (12 wt%)**	THF	0.18–0.40	295-395	9
**Teflon AF2400/PE**	Acetone	1.15	150	10

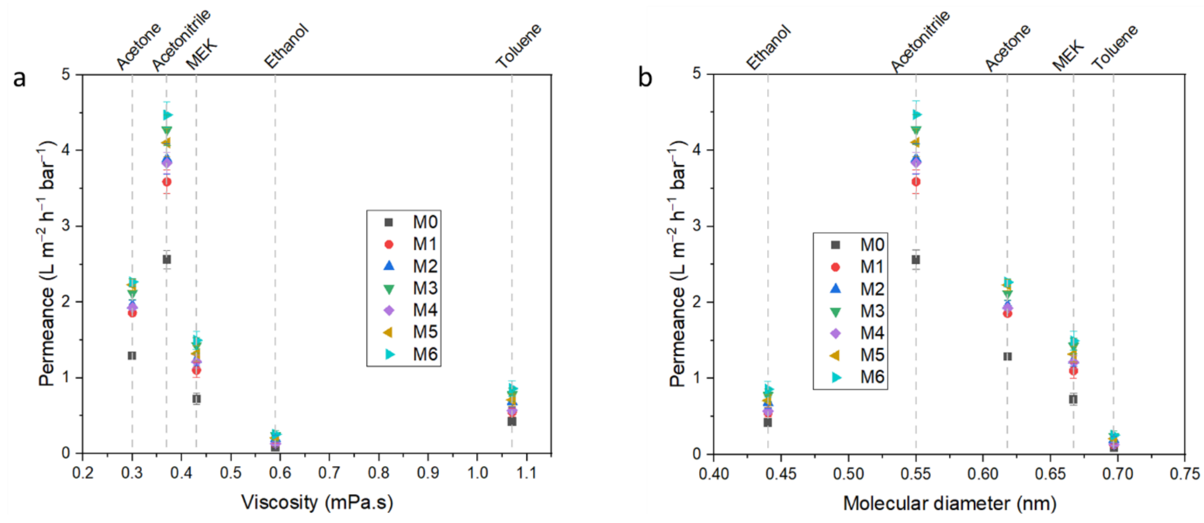

Figure S8. Correlation between solvent's viscosity and permeance (a), and between solvent's molecular diameter and permeance.

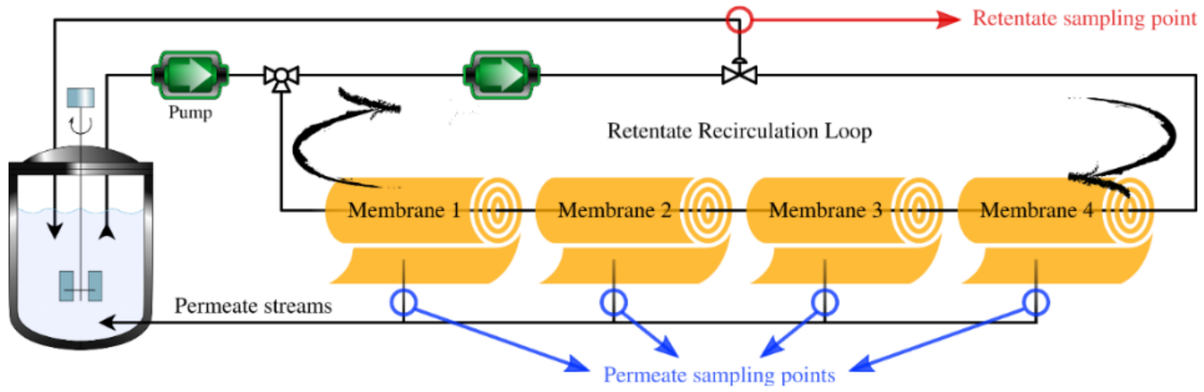

Figure S9. Schematic of the multistage crossflow nanofiltration apparatus used for membrane testing. Note that flat sheet membranes were used.

3. Pore-size calculation

As suggested by Livingston *et al.*, a solvent's permeance can be correlated to its physical properties.[2]:

$$d_{\mathrm{m}} = 2 \cdot \left(\frac{3V_{\mathrm{m}}}{4\pi N_{\mathrm{A}}}\right)^{\frac{1}{3}}, \tag{S1}$$

where V_{m} is the molar volume obtained from the solvent density and N_{A} is the Avogadro's number. The Hagen–Poiseuille equation defines the volumetric flux (J_{v}) through a membrane comprising uniform capillaries.

$$J_{v,i} = \frac{r_i^2 \Delta P \varepsilon}{8\mu_0 l}, \tag{S2}$$

where ε is the porosity, ΔP is the transmembrane pressure, l is the capillary length, μ_0 is the solvent bulk viscosity, and r_{i} is the capillary radius. Next, the pore flow rate ($Q_{p,i}$) allows the calculations of the flow through a pore with radius r_i.

$$Q_{p,i} = \frac{\pi r_i^4 \Delta P}{8\mu_0 l}. \tag{S3}$$

The overall solute rejection can be calculated using the following set of equations:

$$R_{ij} = 1 - \frac{\Phi_{ij} K_{c,ij}}{1 - \left(1 - \Phi_{ij} K_{c,ij}\right)\left(\exp(-P_{e,ij})\right)}, \tag{S4}$$

where Φ_{ij} is a partition coefficient and λ_{ij} is a ratio between the solute radius ($r_{s,j}$) (the subindex for a solute is j) and pore radius (r_i) (the subindex for a pore-size class in the discrete method is i).

$$\Phi_{ij} = \left(1 - \lambda_{ij}\right)^2. \tag{S5}$$

$$\lambda_{ij} = \frac{r_{s,j}}{r_i}. \tag{S6}$$

Assuming a steric interaction between the solute and pore wall, the solute convective, $K_{c,ij}$, and diffusive hindrance, $K_{d,ij}$, factors are expressed as follows:

$$K_{c,ij} = \left(2 - \Phi_{ij}\right)\left(1 + 0.054\lambda_{ij} - 0.988\lambda_{ij}^2 + 0.44\lambda_{ij}^3\right). \tag{S7}$$

$$K_{d,ij} = 1 - 2.3\lambda_{ij} + 1.154\lambda_{ij}^2 + 0.224\lambda_{ij}^3. \tag{S8}$$

The Peclet number ($P_{e,ij}$) characterizing the pore flow is defined as

$$P_{e,ij} = \frac{K_{c,ij}}{K_{d,ij} D_{s,j}}\left(\frac{r_i^2 \Delta P}{8\mu_{p,i}}\right). \tag{S9}$$

The diffusivity. $D_{s,ij}$, of a solute with radius $r_{s,j}$ is calculated using the Stokes–Einstein equation.

$$D_{s,ij} = \frac{kT}{6\pi\mu_{p,i} r_{s,j}} , \tag{S10}$$

where k is the Boltzmann constant and T is the temperature. The Wilke–Chang formula can be used to solve the above equation and estimate the solute diffusivity.

$$D_{s,ij} = 7.4 \times 10^{-8} \frac{T\sqrt{\phi M_{solv}}}{\mu_{p,i} V_{m,j}^{0.6}} , \tag{S11}$$

where M_{solv} is the molecular weight of the solvent molecule, ϕ is a dimensionless solvent parameter, and $V_{m,j}$ is the solute molar volume (in $cm^3 \cdot g \cdot mol^{-1}$). If the rejection $R(r)$ is a continuous function of the pore radius r, the probability density function, $f_R(r)$, is introduced to describe the pore-size distribution.

$$f(r) = \frac{1}{r\sqrt{2\pi b}} \exp\left[-\frac{\left(\log(r/r^*) + b/2\right)^2}{2b} \right] . \tag{S12}$$

$$b = \log\left[1 + \left(\frac{\sigma}{r^*} \right)^2 \right] \tag{S13}$$

For calculating function $f(r)$, the mean pore radius (r^*) and the standard deviation (σ) must be estimated. For simplification, the distribution function is truncated to $r_{\max}$.

$$\frac{f'_R(r)}{f_R(r)} = \frac{1}{\int_0^{r_{\max}} f_R(r)dr} \tag{S14}$$

The overall rejection over the pore radii $0 < r < r_{\max}$ can be calculated using the following expression:

$$R_j = \frac{\int_0^{r_{\max}} f'_R(r) r^4 R(r) / \mu(r) dr}{\int_0^{r_{\max}} f'_R(r) r^4 / \mu(r) dr} . \tag{S15}$$

Implementing the above models, the mean pore size and standard deviation can be fitted by minimizing the error.

4. References

1. Abdulhamid, M. A., Hardian, R. & Szekely, G., Appl. Mater. Today, 28, (2022), 101541. https://doi.org/10.1016/j.apmt.2022.101541
2. Karan, S., Jiang, Z. & Livingston, A. G., Science 348, (2015), 1347–1351. DOI: 10.1126/science.aaa5058
3. Y.H. See-Toh, F.C. Ferreira, A.G. Livingston, J. Membr. Sci., 299 (2007) 236-250. https://doi.org/10.1016/j.memsci.2007.04.047
4. Y.H. See-Toh, M. Silva, A. Livingston, J. Membr. Sci., 324 (2008) 220-232. https://doi.org/10.1016/j.memsci.2008.07.023
5. Y.H.S Toh, F.W. Lim, A.G. Livingston, J. Membr. Sci., 301 (2007) 3-10. https://doi.org/10.1016/j.memsci.2007.06.034
6. S. Darvishmanesh, J. Degrève, B. V. der Bruggen, ChemPhysChem, 11 (2010) 404-411. https://doi.org/10.1002/cphc.200900641
7. A.A. Tashvigh, T.S. Chung, J. Membr. Sci., 572 (2019) 580-587. https://doi.org/10.1016/j.memsci.2018.11.048
8. M.H.D.A. Farahani, T.S. Chung, Sep. Purif. Technol. 209 (2019) 182-192. https://doi.org/10.1016/j.seppur.2018.07.026
9. J. da Silva Burgal, L. Peeva, .P Marchetti, A. Livingston, J. Membr. Sci., 493, (2015) 524-538. https://doi.org/10.1016/j.memsci.2015.07.012
10. G.M. Shi, T.S. Chung, J. Membr. Sci., 602 (2020) 117972. https://doi.org/10.1016/j.memsci.2020.117972
